# Gender discrimination and personal and professional development fostered by allopathic medical schools in the United States

**DOI:** 10.1371/journal.pone.0319549

**Published:** 2026-06-22

**Authors:** Shruthi Venkataraman, Mytien Nguyen, Sarwat I. Chaudhry, Mayur M. Desai, Tonya L. Fancher, Alexandra M. Hajduk, Hyacinth R. C. Mason, Alexis Webber, Dowin Boatright

**Affiliations:** 1 Department of Emergency Medicine, New York University Grossman School of Medicine, New York, New York, United States of America; 2 Department of Immunobiology, Yale School of Medicine, New Haven, Connecticut, United States of America; 3 Section of General Internal Medicine, Department of Internal Medicine, Yale School of Medicine, New Haven, Connecticut, United States of America; 4 Department of Chronic Disease Epidemiology, Yale School of Public Health, New Haven, Connecticut, United States of America; 5 Division of General Internal Medicine, Department of Internal Medicine, University of California, Davis, School of Medicine, Sacramento, California, United States of America; 6 Section of Geriatrics, Department of Internal Medicine, Yale School of Medicine, New Haven, Connecticut, United States of America; 7 Office of Student Affairs and Department of Family Medicine, Tufts University School of Medicine, Boston, Massachusetts, United States of America; 8 Section on Gerontology and Geriatric Medicine, Department of Internal Medicine, Wake Forest University School of Medicine, Winston Salem, North Carolina, United States of America; University of Oxford, UNITED KINGDOM OF GREAT BRITAIN AND NORTHERN IRELAND

## Abstract

**Background:**

Despite prevalent gender discrimination in medical education, its influence on personal and professional development, foundational competencies in medical training per the Association of American Medical Colleges (AAMC), remains unclear. This retrospective cross-sectional study assesses how experiences of gender discrimination in medical school influence personal and professional identity formation (PPIF) among males and females.

**Methods:**

Deidentified student-level data were procured from the AAMC data warehouse for 37,610 MD students who matriculated in 2014–2015 and took the Graduation Questionnaire (GQ) between 2016–2020. Gender discrimination frequency was categorized as ‘Never’, ‘Isolated’, and ‘Recurrent’ from GQ responses to questions about denial of opportunities, offensive remarks, and lower evaluations due to gender. Students self-reported their sex as male, female or declined to answer. PPIF was assessed using two separate GQ metrics assessing student agreement on a 5-point Likert scale that their medical school fostered and nurtured their development as a person and a future physician, respectively, and dichotomized.

**Results:**

Female students experienced higher rates of isolated (12.6%) and recurrent (20.1%) gender discrimination than males (4.3% isolated, 6.2% recurrent). Females reported slightly lower personal (71.2%) but similar professional development (92.2%) rates compared to males (73.4% personal, 91.2% professional). Both sexes experiencing gender discrimination had lower likelihoods of PPIF than their counterparts without these experiences. If recurrent discrimination occurred, the aRR (95%CI) of professional development was 0.89 (0.87–0.90) for females and 0.78 (0.74–0.81) for males, while for personal development, it was 0.69 (0.67–0.71) for females and 0.61 (0.58–0.66) for males. Compared to females, males showed sharper declines in professional development as discrimination frequency increased from never to isolated (aRR = 0.93, 95% CI [0.92–0.94], p < 0.001) and isolated to recurrent (aRR = 0.95, 95% CI [0.93–0.97], p < 0.001).

**Conclusions:**

Gender discrimination negatively influences PPIF for both female and male medical students. Efforts to combat discrimination in medical training and promote holistic student development should be considered. Future work is needed to understand the influence of gender discrimination on the comprehensive development of gender-diverse medical students.

## Introduction

Gender-based bias and discrimination remain pervasive in medical training, with far-reaching implications for medical students’ development. Studies conducted in different contexts underscore that this problem is widespread yet manifests in varied ways. For example, a 14-school survey in the United States found that 93% of female and 83% of male students had encountered at least one incident of gender discrimination or harassment during medical school [1]. Nearly half of those women reported that these negative experiences influenced their choice of specialty, compared to 16% of affected men [1]. Similarly, a recent survey in Germany documented frequent gender bias: 88% of women reported experiencing gender discrimination, versus 46% of men [2]. Notably, one in ten female respondents in that study said they avoided or ruled out certain specialties entirely due to anticipated gender-based bias, a striking indication of how deeply these perceptions can shape career trajectories [2]. In a 1995 report, the Council on Graduate Medical Education in the United States stated: “Gender bias, a reflection of society’s value system, remains the single greatest deterrent to women achieving their full potential in every aspect of the medical profession and is a barrier throughout the professional life cycle” [1].

Importantly, gender discrimination in medical school is not always overt; it often operates through subtle “hidden curriculum” signals. Qualitative research in the UK and Scandinavia describes how everyday interactions – from gender‐stereotyped expectations and offhand remarks to the dearth of female role models in certain fields – convey implicit messages about who belongs where [2,3]. Female students report having to work harder to “fit in,” sometimes altering their behavior or appearance to navigate gendered expectations [4]. At the same time, context matters. In traditionally female-dominated fields like obstetrics and gynecology, male medical students have reported feeling marginalized because of their gender [1,4]. Even as blatant sexism and harassment have become less socially acceptable over time, research suggests that implicit bias and structural inequities persist. Physical symbols of institutional culture (such as walls filled with portraits of male leaders) and lingering pay and promotion gaps send a message that medicine is still “too male, too pale… too stale,” as one UK study put it [3]. In short, despite progress, gender inequality remains woven into the fabric of medical education across diverse contexts, reinforcing the need to understand its impact on learners.

One critical gap in the literature – and the focus of our study – is how experiences of gender discrimination influence medical students’ personal and professional identity formation (PPIF). Professional identity formation is the process by which trainees internalize the values, behaviors, and sense of belonging to the medical profession. The Association of American Medical Colleges (AAMC) emphasizes the importance of personal and professional development—cultivating qualities necessary for lifelong growth as a person and a physician—as foundational competencies in medical training [5]. The learning environment in medical school shapes identity formation and is influenced by interactions with peers, patients, and supervisors [6–8].

While the existence of gender discrimination in medical school has been documented, the “full spectrum” of gender-based experiences and its specific effect on students’ emerging identities as physicians is underexplored [9]. Emerging evidence, however, suggests the effect is significant. Babaria et al. observed that third-year female students become acculturated to unprofessional behavior from male supervisors: encountering frequent sexism, they described feelings of guilt and diminished self-confidence but, with time, grew “used to it” and resigned to the idea that such mistreatment was simply part of being a woman doctor [9]. Similarly, a focus-group study from Sweden – a country known for its commitment to gender equality – found that both female and male students adjusted their behavior to cope with a gendered clinical “climate” [4]. Students reported staying silent or avoiding challenging unprofessional behavior from supervisors due to fear of repercussions, indicating how power dynamics and sexism pushed them to conform rather than speak up [4]. Such informal lessons in medical school (the hidden curriculum) have a powerful influence on socialization and identity formation [10]. They can subtly shape how future physicians see themselves and their role in the profession, for example by reinforcing doubts about belonging or steering individuals away from certain identities (e.g., surgeon, leader) that seem “off-limits” to their gender [3].

Prior studies examining gender bias in medical education have largely been qualitative and conducted in a select number of institutions. The context of the present study helps address these gaps by examining the influence of graded gender discrimination experience on medical schools’ ability to foster personal and professional identity formation among male and female medical students in the United States using national quantitative data. This work will shed light on an under-addressed aspect of medical training (the influence of gender discrimination on identity formation), thereby helping educators and policymakers better understand how to foster an inclusive learning environment that supports all students’ holistic growth.

## Methods

### Data and participants

Using fully anonymized student-level data from the AAMC data warehouse, we conducted a retrospective cross-sectional study of 40,971 Doctor of Medicine (MD) matriculants from the 2014–2015 and 2015–2016 academic years who graduated between 2016 and 2020 and participated in the American Medical College Application Service (AMCAS) survey and Graduation Questionnaire (GQ). Students provided explicit electronic consent on AMCAS by actively selecting an option to allow the use of their data for research purposes as outlined in AAMC policies. Participation in the GQ was voluntary, and students were informed that their responses could be used for research purposes, providing implied informed consent by choosing to participate. The Yale School of Medicine IRB determined that the parent study, for which the AAMC data used in this study were originally procured, does not constitute human subjects research (IRB Protocol ID: 2000028658). As the AAMC fully deidentified all data before transfer and researcher access, additional ethics review and informed consent were not required for this study. Data were accessed for research purposes between February 1, 2024, and June 3, 2024. The study followed the Strengthening the Reporting of Observational Studies in Epidemiology reporting guidelines for cross-sectional studies. Four students with unknown sex and 3,357 students who did not graduate from medical school were not included in the analysis. The final sample included 37,610 participants.

### Student sociodemographic characteristics

Data on sex, race and ethnicity, and childhood household income were obtained from the AMCAS. Students self-reported their sex as male, female, or ‘decline to answer’. The item was labeled ‘Sex’ on the 2014 and 2015 AMCAS application and was not accompanied by a definition or clarification about whether it referred to sex assigned at birth or gender identity. As such, this variable may reflect sex, gender identity, or a combination thereof, depending on how applicants interpreted the question. We therefore interpret analyses as stratified by the AMCAS ‘Sex’ response and acknowledge potential nondifferential misclassification. Students self-reported their race and ethnicity as corresponding to any or all the following groups: African American or Black, American Indian or Alaska Native (AIAN), Asian, Hawaiian Native or Pacific Islander (HNPI), Hispanic, Latino, or of Spanish Origin, white, other, and unknown. Students reporting identification with 2 or more groups were categorized as multiracial. Students self-selected their childhood household income from 17 categories, which were then recategorized into 5 ranges to approximate income quintiles: less than $50,000, $50,000 to less than $75,000, $75,000 to less than $125,000, $125,000 to less than $200,000 and $200,000 or more.

### Experience of gender discrimination

Gender discrimination frequency was determined via the following three questions in the GQ: 1) “During medical school, how frequently have you been denied opportunities for training or rewards based on gender?” 2) “During medical school, how frequently have you been subjected to sexist remarks/names?” and 3) “During medical school, how frequently have you received lower evaluations or grades solely because of gender rather than performance?” To each question, participants could respond ‘Never’, ‘Once’, ‘Occasionally’ or ‘Frequently’. We recategorized the variable into three levels: none, isolated, and recurrent. Participants who reported ‘None’ to all three questions were categorized as having no experiences of gender discrimination. Those who responded ‘Once’ to only one of the questions were categorized as having isolated experiences. Participants were classified as having recurrent experiences if they met one of the following criteria: at least one response of ‘Occasionally’ or ‘Frequently’ to any question, or ‘Once’ to more than one question. These three items are regularly included in the annual AAMC Graduation Questionnaire and are the most widely available national-level indicators of gender-based mistreatment in U.S. allopathic medical education. These items have been psychometrically validated as part of the PRODIGIE tool using the same 2016–2020 GQ cohorts analyzed in our study, undergoing exploratory and confirmatory factor analyses and demonstrating acceptable internal consistency for the discrimination factor (Cronbach α = 0.76) [11]. While these items were validated within the broader discrimination factor in PRODIGIE, we use them here as a formative index capturing distinct manifestations of gender‑based mistreatment, for which high internal consistency is not required. When assessed in our sample, the three items demonstrated a Cronbach’s α of 0.58, which is expected for a brief formative index. The same AAMC GQ items have been used in multiple national studies examining discrimination among U.S. medical students [12,13].

### Outcomes

PPIF was assessed using two distinct metrics: personal and professional development. Personal development was measured by the GQ item “My medical school has done a good job of fostering and nurturing my development as a person” and professional development was measured by the GQ item “My medical school has done a good job of fostering and nurturing my development as a physician”. Responses were recorded on a 5-point Likert scale from ‘Strongly disagree’ to ‘Strongly agree’. Variables were dichotomized such that only participants who expressed strong agreement (i.e., ‘Agree’ or ‘Strongly agree’) were considered as reporting that their medical school nurtured their PPIF. Both personal and professional development were examined separately in all analyses; the term PPIF is used to collectively refer to these two outcomes. The two outcome items originated from a single-institution study at Wake Forest University in 1996 [14] and were subsequently adopted into the AAMC GQ. While they are not components of a validated scale and cannot be tested for reliability as single items, they are used in national research and institutional evaluation as global indicators of perceived institutional support for personal and professional development [15–18].

### Statistical analysis

All data analyses were conducted from December 1, 2023, to July 12, 2024, using Stata version 16.1 (StataCorp LLC). Missing data were present across several key variables, with the extent of missingness varying. While there were no missing data for binary sex, 6.46% of the data were missing for race/ethnicity, 22.43% of the data were missing for childhood household income, 27.5% of data were missing for experience of gender discrimination, 20.6% for personal development, and 19.8% for professional development. A total of 21,728 participants (57.77%) answered all the questionnaire items relevant to this study. Missing data were imputed using a fully conditional specification method to handle arbitrary missing patterns across all categorical data. The multiple imputation model included all predictors and outcomes as well as indicators plausibly related to missingness. Twenty imputed data sets were created. We assumed data were missing at random (MAR) conditional on included variables. Diagnostics demonstrated model convergence and close alignment between observed and imputed marginal distributions (available on request). Complete-case analyses (n = 21,728) yielded estimates similar in direction and magnitude (available on request). We present the imputed results to reduce potential bias due to listwise deletion and to retain statistical power.

To investigate the independent effect of sex on the PPIF outcomes, we utilized generalized linear models (GLMs) with a Poisson distribution and robust standard errors wherein sex or experience of gender discrimination were independent variables and personal and professional development were the outcome variables. We used this method because PPIF outcomes were common [19,20]. To examine sex differences in the association between gender discrimination and PPIF, we used two complementary modeling approaches. First, we created a composite 6-level categorical variable representing all combinations of sex (male/female) and discrimination frequency (never, isolated, recurrent), which we used as the main predictor in Poisson regression models with robust standard errors to estimate adjusted relative risks for each group. Second, to formally test for interaction, we specified a Poisson regression model that included a cross-product term between sex and discrimination frequency. We used post-estimation margins to calculate adjusted predicted probabilities and applied linear contrasts to evaluate whether the magnitude of change in outcome probabilities across increasing discrimination levels differed by sex. Two-sided *p*  <  .05 indicated significance. All relative risk estimates are adjusted for race, ethnicity, and childhood household income. Sensitivity analyses including indicator terms for GQ year were conducted to assess potential cohort effects.

## Results

The imputed dataset (N = 37,610) showed a slightly lower proportion of females (48.4%) than males (Table 1). Over 20% of participants reported experiencing gender discrimination, with 12.9% experiencing it recurrently. Most students reported that their medical schools fostered their personal (72.3%) and professional (91.7%) development, with a notably higher endorsement of professional development.

**Table 1:** Participant demographic characteristics.

Characteristic	Imputed Dataset No. (%) N = 37,610
Sex	
Female	18,200 (48.4)
Race/Ethnicity	
African American or Black	2460 (6.5)
Asian	7810 (20.8)
Hispanic	2438 (6.5)
multiracial	2410 (6.4)
white	21,355 (56.8)
other^1^	1137 (3.0)
Yearly Childhood Household Income	
<$50,000	7983 (21.2)
$50,000 to <$75,000	6321 (16.8)
$75,000 to <$125,000	11,184 (29.7)
$125,000 to <$200,000	6075 (16.2)
≥ $200,000	6047 (16.1)
Experience of Gender Discrimination	
Never	29,622 (78.8)
Isolated	3129 (8.3)
Recurrent	4859 (12.9)
Personal Development	
Yes	27,195 (72.3)
Professional Development	
Yes	34,488 (91.7)

^1^American Indian/Alaska Native and Hawaiian Native/Pacific Islander students were included as part of the ‘other’ group, due to their small sample size.

### Prevalence of gender discrimination among female and male students

**Fig 1:**
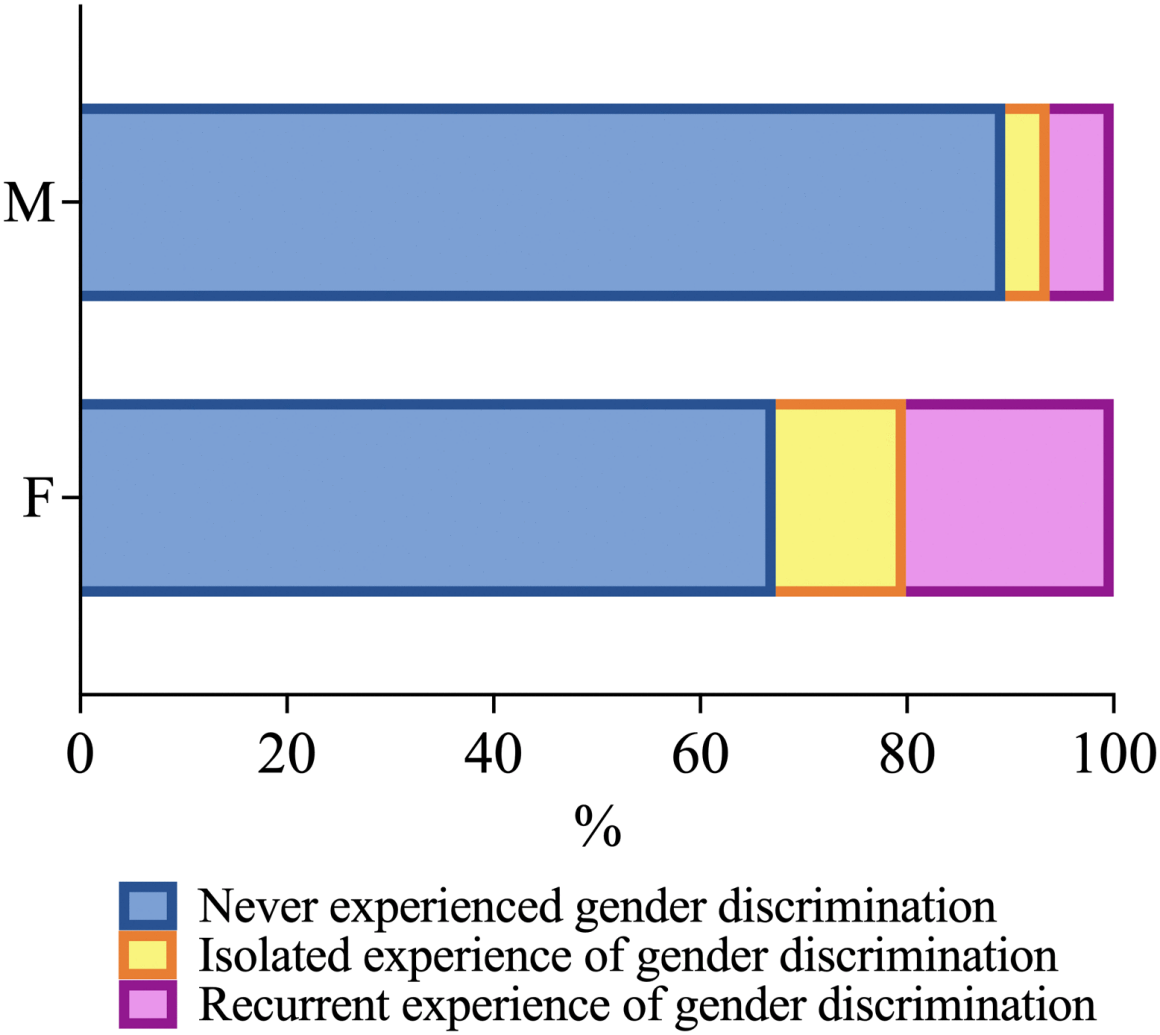
Frequency of gender discrimination experienced by male (M) and female (F) medical students. The stacked bar charts display the percentages of male and female medical students who experienced gender discrimination. The data is categorized into three groups: those who never experienced gender discrimination (blue), those who had isolated experiences of gender discrimination (yellow), and those who had recurrent experiences of gender discrimination (pink).

Among females, 20.1% (N = 3658) experienced recurrent, 12.6% (N = 2293) experienced isolated, and 67.3% (N = 12,249) never experienced gender discrimination (Fig 1). Among males, 6.2% (N = 1203) experienced recurrent, 4.3% (N = 835) experienced isolated, and 89.5% (N = 17,372) never experienced gender discrimination. Experiences of gender discrimination were significantly more common among female students compared to their male counterparts (χ^2^(2)=2769.4, p < 0.001).

### Influence of binary sex on PPIF in medical education

**Fig 2:**
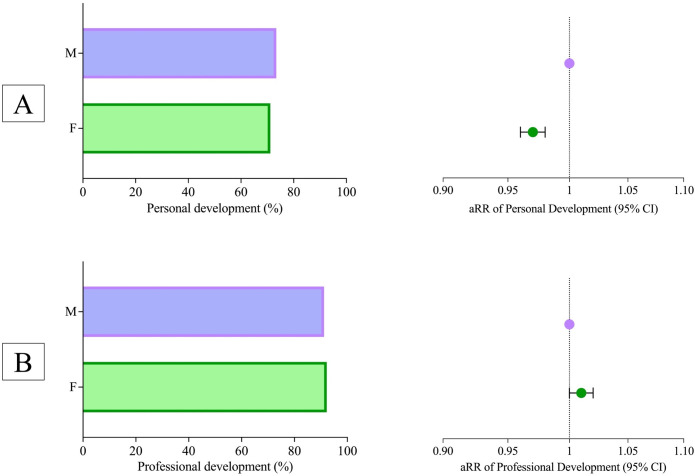
Personal and Professional Development Among Medical Students by Sex. Panel A shows the proportion of female (F) and male (M) medical students reporting that their medical school fostered their personal development. The associated forest plot on the right illustrates the adjusted relative risk (aRR) for personal development with 95% confidence intervals, comparing female students to their male counterparts (reference group). Panel B shows the proportion of female and male medical students reporting that their medical school fostered their professional development. The associated forest plot on the right illustrates the aRR for professional development with 95% confidence intervals, comparing female students to their male counterparts. All relative risks were adjusted for race/ethnicity and childhood income.

Female students were less likely to report that their medical school fostered and nurtured their personal development (71.2%, N = 12,950) than male students (73.4%, N = 14,246, χ^2^(1)=23.57, p < 0.001, Fig 2A). Compared to males, females reported a lower likelihood of their medical school fostering their personal development (adjusted Relative Risk; aRR = 0.97, 95% CI 0.96–0.98). Female students were more likely to report that their medical school fostered and nurtured their professional development (92.2%, N = 17,705) than male students (91.2%, N = 16,796, χ^2^(1)=1430.75, p < 0.001, Fig 2B). However, the likelihood of agreeing that their medical school fostered their professional development was not significantly different for females compared to males after adjustment for race/ethnicity and childhood household income (aRR = 1.01, 95% CI 1.00–1.02).

### Influence of gender discrimination on PPIF in medical education among female and male students

**Fig 3:**
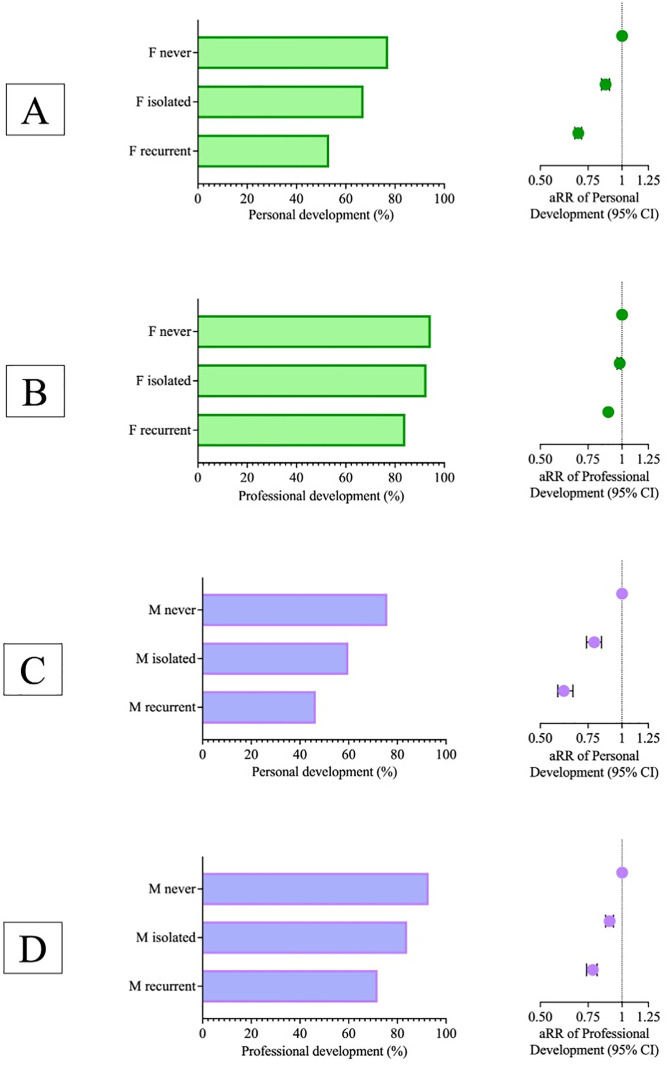
Personal and Professional Development Among Female and Male Medical Students by Frequency of Gender Discrimination. Panels A and C show horizontal bar charts depicting the percentages of personal development for females and males who never experienced, had isolated experiences of, or recurrent experiences of gender discrimination, respectively. The associated forest plots on the right presents the adjusted relative risk (aRR) of personal development for females or males with 95% confidence intervals, based on the level of gender discrimination experienced. Panels B and D show horizontal bar charts depicting the percentages of professional development for females and males who never experienced, had isolated experiences of, or recurrent experiences of gender discrimination, respectively. The associated forest plots on the right presents the aRR of professional development for females or males with 95% confidence intervals, according to the level of gender discrimination experienced. All relative risks were adjusted for race/ethnicity and childhood income.

Among females, the prevalence and likelihood of students reporting that their medical school fostered their personal development was inversely proportional to the frequency of gender discrimination: 77.2% (N = 9,443, reference for aRR) if none, 67.3% (N = 1,549, aRR = 0.87, 95%CI 0.84–0.90) if isolated, and 53.3% (N = 1,957, aRR = 0.69, 95%CI 0.67–0.71) if recurrent (χ^2^(2)=788.37, p < 0.001, Fig 3A). Patterns for professional development were similar: 94.5% (N = 11,565, reference for aRR) if none, 92.8% (N = 2,138, aRR = 0.98, 95%CI 0.96–0.99) if isolated, and 84.2% (N = 3,094, aRR = 0.89, 95%CI 0.87–0.90) if recurrent (χ^2^(2)=397.19, p < 0.001, Fig 3B).

Among males, the prevalence and likelihood of students reporting that their medical school fostered their personal development was inversely proportional to the frequency of gender discrimination: 75.9% (N = 13,185, reference for aRR) if none, 59.9% (N = 504, aRR = 0.79, 95%CI 0.74–0.84) if isolated, and 46.5% (N = 557, aRR = 0.61, 95%CI 0.58–0.66) if recurrent (χ^2^(2)=565.83, p < 0.001, Fig 3C). Patterns for professional development were similar: 92.9% (N = 16,145, reference for aRR) if none, 84% (N = 703, aRR = 0.90, 95%CI 0.87–0.93) if isolated, and 71.9% (N = 857, aRR = 0.78, 95%CI 0.74–0.81) if recurrent (χ^2^(2)=680.91, p < 0.001, Fig 3D).

### Sharper declines in PPIF rates for males with increasing frequency of gender discrimination

**Fig 4:**
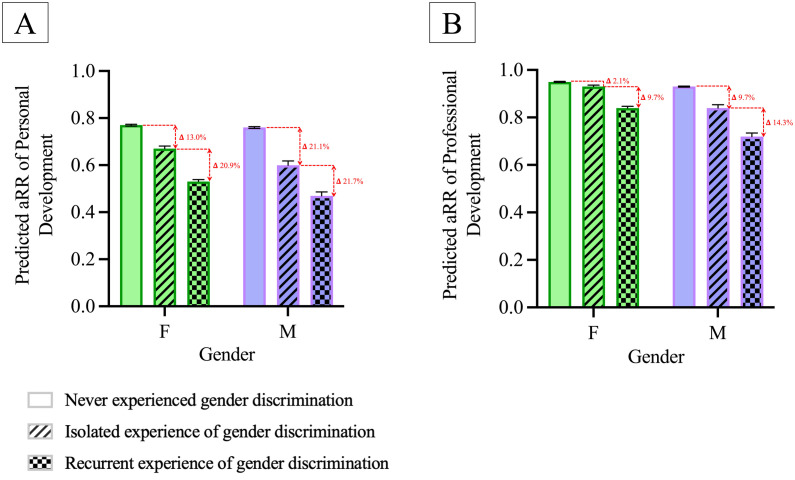
Predicted Personal and Professional Development in Males and Females by Frequency of Gender Discrimination. Panel A illustrates the predicted adjusted relative risk (aRR) of personal development for female (green) and male (purple) medical students based on their experience of gender discrimination (never, isolated, or recurrent). Panel B shows the predicted aRR of professional development for female (green) and male (purple) medical students by their experience of gender discrimination (never, isolated, or recurrent). For both panels, the red arrows and corresponding delta (Δ) values represent the percentage decrease in aRR as the frequency of reported gender discrimination increases from no discrimination to isolated discrimination, and from isolated to recurrent discrimination, for both males and females. All predicted relative risks are adjusted for race/ethnicity and childhood household income.

The change in predicted relative risk of the school fostering personal development among students who never experienced gender discrimination compared to students who experienced isolated discrimination was significantly higher in male students than female students (aRR = 0.90, 95% CI 0.88–0.92, p < 0.001, Fig 4A). The change in predicted relative risk of the school fostering personal development among students who experienced isolated gender discrimination compared to those who experienced recurrent discrimination was not significantly different between males and females.

The change in predicted relative risk of the school fostering professional development among students who never experienced gender discrimination compared to students who experienced isolated discrimination was significantly higher in males than females (aRR = 0.93, 95% CI 0.92–0.94, p < 0.001, Fig 4B). The change in predicted relative risk of the school fostering professional development among students who experienced isolated gender discrimination compared to students who experienced recurrent discrimination was significantly higher in males than females (aRR = 0.95, 95% CI 0.93–0.97, p < 0.001). Across analyses, adding GQ-year indicator terms in sensitivity analyses produced results consistent with the main models.

## Discussion

This study uncovers significant, progressive, and consistent links between experiences of gender discrimination and the lack of personal and professional development fostered by medical schools among their male and female students. While our study examines binary sex-based differences, the measure may also reflect participants’ gender identity. We therefore contextualize our findings with prior research examining gender differences, which appears to be the predominant focus in medical education literature. Although previous research has highlighted the existence of gender discrimination in undergraduate medical education [9,21,22], to our knowledge, this is the first study to investigate its influence on shaping medical student PPIF using national data.

### Female students reported more gender discrimination than males

As expected, our study indicates that female students report recurrent and isolated experiences of gender discrimination more frequently than their male counterparts. Women often face more adverse experiences in men-dominated fields like medicine, including stereotyping and bias [23,24], and disparaging comments related to their gender, gender discrimination, and sexual harassment [25]. These experiences can be attributed to societal and institutional norms that have historically favored men in medical professions [24]. Critically, over one-third of female medical students in our study experienced gender discrimination and over one-fifth experienced it recurrently. These frequent experiences of discrimination have been attributed to diminished career aspirations among women medical trainees [1], as well as negative impacts on self-esteem [26], self-efficacy [27] and mental health [28], likely impeding their PPIF. Not only do female students face significantly more gender discrimination than males, but this inequity persists into their careers, as evidenced by female faculty who also report higher rates of discrimination compared to their male counterparts [29]. Possibly due to the continued experience of gender discrimination, female faculty experience lower career satisfaction [30,31], higher burnout rates [32], less research productivity [25], lower earnings [25] and slower career advancement [25], and hold far fewer full professorships (29%) and departmental chair positions (24%) [33]. Similarly, among female medical students, gender discrimination may impede PPIF by contributing to burnout [34], and diminishing opportunities for leadership and professional growth [1]. Addressing gender discrimination early in medical education might help mitigate the long-term impacts on female students.

### Female students reported similar professional but lower personal development than male students

The AAMC identifies personal and professional development as key competencies in medical education [5]. Our study indicates that both females and males are equally likely to attest that their schools fostered their professional development, possibly reflecting an appropriately increasing focus on gender inclusivity in medical education in recent times [35]. Females reporting similar rates of professional development despite experiencing higher rates of discrimination perhaps suggests that they may employ resilience and coping strategies that buffer against the adverse effects of gender discrimination on professional aspirations [36]. Our study also suggests that female medical students perceive a lack of support for their personal development from their medical schools compared to their male counterparts. This discrepancy may stem from underlying systemic biases and gender-specific challenges that women face in medical education [9]. Studies have shown that the gender discrimination and microaggressions that female students often encounter in the learning environment can hinder their sense of belonging and support within the academic environment [9,22].

### The influence of gender discrimination on development differed by sex

In our study, with progressively greater levels of gender discrimination, all students report lower PPIF. Gender discrimination in educational environments has been shown to negatively impact educational outcomes for both females and males [37], contributing to decreased academic performance [38], and biased evaluations [37]. Qualitative research suggests that gender discrimination in medical school shapes professional identity formation among females by restricting access to meaningful participation, recognition, and support in clinical training environments [3,39,40]. Across studies, women reported being perceived as less capable, subjected to higher performance thresholds, or overlooked for key learning opportunities, which disrupted their sense of belonging, narrowed their imagined futures in medicine, and in some cases influenced specialty choice [3,39,40]. Some described feeling unequipped to respond to inappropriate behavior from male supervisors, leading to emotional withdrawal, self-blame, and normalization of these experiences as part of becoming a physician [3,9]. The absence of relatable role models and fear of speaking up further limited how women could safely reflect on or challenge these experiences [9,39,40]. These processes were reinforced by gendered double standards, where women were penalized both for lacking confidence and for displaying it, revealing how professional legitimacy was tethered to conflicting and exclusionary norms [3,39]. Interestingly, our study observes that gender discrimination is associated with a greater negative influence on PPIF in males compared to females. While the mechanisms underlying this pattern cannot be determined from our data, we interpret these findings cautiously in light of prior empirical and theoretical work. This finding aligns with other research reporting a greater influence of gender discrimination on career decisions among men than women, despite fewer men experiencing gender discrimination, suggesting that men may weigh experiences of discrimination more heavily in their career decisions [1]. This may be because men experience gender discrimination as a more significant violation of social norms, which could lead to greater psychological impact and disruption in their development process [41]. This is consistent with the concept of stereotype threat, where being in the minority or facing unexpected social discrimination can erode self-identity and performance [42]. Men may have fewer coping mechanisms for managing gender discrimination than women, who, due to more frequent exposure, may be more resilient, potentially exacerbating gender discrimination’s negative effects on the PPIF of men. It is also possible that the gender discrimination experienced by men is qualitatively different, perhaps due to fewer support avenues available for men in medicine to discuss these experiences compared to women. However, little qualitative research has examined how men, particularly men of color, experience gender discrimination in medical education, despite the possibility that their experiences may be shaped by intersecting racial and gendered dynamics that differ from those of white men or women [43]. This gap limits our ability to fully interpret the stronger declines in PPIF observed among men, particularly in understanding how men perceive, experience, and respond to gender discrimination, and highlights the need for intersectional qualitative research that captures the experiences of men, women, and gender-diverse students across intersectional identities.

### Gender discrimination influenced personal development more than professional development

Among both females and males, gender discrimination appears to impact personal development more severely than professional development. This difference may be due to the explicit focus of medical education on developing clinical competence and professional behaviors, as delineated by the Liaison Committee on Medical Education (LCME). Notably, the LCME accreditation guidelines do not explicitly address personal development. Including this in their standards could serve as another intervention to enhance holistic PPIF among students facing gender discrimination. Given the influence of gender discrimination on multiple factors influencing personal development, including feelings of isolation, professional self-confidence and satisfaction, self-esteem, collegiality [44], and specialty choice and residency program selection [1], medical schools should consider specific efforts to foster student personal development among students experiencing gender discrimination.

### Applicability to other contexts

Our study draws on data from U.S. medical schools and may have limited generalizability to medical educational settings outside North America where sociocultural norms, gender dynamics, and institutional structures differ and may shape both the experience and perceived impact of gender discrimination in distinct ways. However, there is evidence that gender discrimination also influences PPIF among medical students in other national contexts. In Brazil, a large survey of medical students found that experiences of gender discrimination among cisgender women and nonbinary individuals were associated with lower career satisfaction, diminished self-confidence, increased burnout, and limited access to mentorship, all of which can influence professional identity formation [45]. In Japan, a qualitative study of female physicians reflecting on their training experiences found that gender stereotyping disrupted the integration of personal and professional identities, complicating the development of a stable professional identity [46]. These studies support the broader relevance of our findings and underscore the need for more cross-national research to examine how these dynamics unfold in diverse educational and cultural environments.

## Limitations

Our binary sex variable (female/male) does not capture intersex variations. For our study years, the AAMC did not release data on intersex, non-binary, or other gender-diverse individuals because of the small sample sizes and concerns about maintaining anonymity. Although we refer to this variable as ‘sex’ to match the instrument label, the AMCAS item lacked a definition, which may have led some respondents to interpret it as gender identity, introducing potential misclassification that is likely nondifferential with respect to the outcomes and would bias associations toward the null. Prior work suggest that, in the absence of clarification, respondents may conflate sex and gender when completing survey items [47]. Similarly, the absence of sexual orientation data for our study years limits our ability to explore how intersecting sexual minority status (lesbian, gay, or bisexual identity), may influence personal and professional development. Together, these data limitations restrict the inclusivity and applicability of our findings and highlight the need for future research that captures a broader spectrum of sex, gender, and sexual orientation in medical education. In addition, high non-response rates for sensitive variables, such as childhood household income and experience of gender discrimination, required us to rely on imputation. While multiple imputation slightly shifted the distribution from a female to a male majority—which could, in theory, attenuate associations—the imputation model included gender, discrimination, and both outcome variables, enabling it to preserve differential outcome patterns by sex. Complete-case and imputed estimates were otherwise similar in direction and magnitude. Further, residual bias may persist if data were not missing at random. For instance, underrepresentation of students experiencing discrimination could lead to conservative estimates of its association with educational outcomes. Furthermore, the GQ captures only a subset of gender discrimination experiences; however, those included are significant and can have serious psychological and professional impacts. While the GQ measures used as proxies for personal and professional development assess institutional success in fostering student growth—perhaps appropriately attributing responsibility to the school—they may not fully capture the extent of development, or lack thereof, experienced by students. Each outcome was derived from a single survey item that, while used nationally as an indicator of institutional support, is not part of a validated multidimensional scale. Consequently, these measures may not encompass the complex, dynamic, and individualized nature of personal and professional identity formation. We also recognize that identity development during medical training likely extends beyond the development fostered by the institution to include individual factors, though institutional efforts remain crucial [6,8]. Moreover, as our outcomes and exposures rely on self-report, recall bias, sex differences in perception or interpretation, and social desirability bias may influence reporting patterns. Finally, given the retrospective cross-sectional design, temporal ordering cannot be established, and reverse causality is possible (i.e., lower perceived development could influence interpretation/reporting of discrimination experiences). Associations should therefore be interpreted as non-causal.

## Implications and recommendations

Our findings highlight a persisting need for systemic reforms in medical education to address gender discrimination that is experienced by both female and male students, and to expand inquiry into how such discrimination intersects with broader axes of marginalization—rooted in racialization, class, and sexuality—to shape PPIF and educational trajectories, including among gender-diverse students. Addressing gender discrimination in the learning environment, as well as racial discrimination, as our previous work suggests, may serve to promote holistic development for all students [17]. We propose considering the integration of PPIF as a critical equity metric and formally including PPIF parity for students of all racial, ethnic, and gender backgrounds in the LCME accreditation standards [17]. In line with global recommendations for gender-transformative health professional education, medical schools should periodically conduct institutional “gender audits” to identify how entrenched gender norms, power hierarchies, and policies—collectively termed gender regimes—shape student experiences and perpetuate inequities [48]. Embedding such audits within accreditation and quality-improvement processes could ensure accountability for sustained gender equity.

Beyond brief stand-alone bias sessions, institutions should implement a coordinated set of actions that operate at individual, organizational, and structural levels. A successful example is Stanford University’s multifaceted gender climate initiative, which required all faculty to attend sexual-harassment prevention training; convened small-group leadership retreats to address gender and racial dynamics in supervision and evaluation; provided student-focused workshops on professional boundaries and reporting pathways; created a standing diversity council reporting directly to the Dean; and formalized clear, accessible procedures for addressing harassment complaints [49]. Building on this model, a systematic review recommended implementation of a “basic bundle” of interventions including (1) explicit institutional policies that clearly define prohibited behaviors and outline sanctions, (2) confidential and well-supported reporting mechanisms with guaranteed follow-up and protection from retaliation, and (3) structured educational programming tailored to different roles—including faculty evaluators, clinical supervisors, and students—to reinforce accountability and behavioral expectations [50]. These reforms should be complemented by measures addressing caregiver discrimination—such as flexible training schedules, protected parental leave, and accessible child-care supports—which have been identified as critical for retaining trainees and sustaining equitable professional identity development [48].

Fostering a sense of belonging for minoritized and marginalized learners in medicine—by uncovering unconscious biases and transforming academic medicine environments to recognize, hear, and value their contributions—can positively influence PPIF in medical school [51,52]. Validated tools that can systematically measure and help achieve a climate of equity and inclusion within the medical school learning environment, such as the PRODIGIE tool developed by our team, may provide medical schools with actionable insights to foster equity and inclusion [11], ultimately improving educational outcomes, including PPIF, for all students. Parallel structural supports—such as dedicated gender-equity or equal-opportunity offices that coordinate harassment prevention, caregiving policies, and climate data reporting—have been identified as foundational components of sustainable gender-transformative reform [48]. Complimentary structural interventions include increasing the representation of women and gender-diverse individuals in academic medicine leadership, and embedding climate and inclusion metrics in faculty promotion criteria [50,53,54].

## Conclusion

Our study provides empirical evidence highlighting the influence of gender discrimination on the effectiveness of medical schools in nurturing students’ PPIF. Female students experience gender discrimination more frequently than their male counterparts. Females were also less likely to report that their medical school fostered their personal development than males. Increasing frequency of gender discrimination correlates with a decreased likelihood, for both females and males, that their school supported their PPIF. However, males experienced sharper declines in PPIF rates with increasing frequency of gender discrimination compared to females. Overall, these findings highlight a potential influence of gender discrimination on development within educational settings, calling for targeted interventions to address gender discrimination in medical school and foster an environment conducive to equitable development for all students. Future research is needed to understand how gender discrimination influences the holistic development of medical students across the spectrum of gender identity and expression—including in more recent national cohorts—and through longitudinal qualitative and mixed methods approaches that include women, men, and gender-diverse students with intersecting marginalized identities.

## Supporting information

S1 Table Female students: Frequency of gender discrimination (corresponds to Fig 1). (DOCX)

S2 Table Male students: Frequency of gender discrimination (corresponds to Fig 1). (DOCX)

S3 Table Personal development by sex (corresponds to Fig 2A). (DOCX)

S4 Table Professional development by sex (corresponds to Fig 2B). (DOCX)

S5 Table Female PPIF by discrimination frequency (corresponds to Fig 3A, B). (DOCX)

S6 Table Male PPIF by discrimination frequency (corresponds to Fig 3C, D). (DOCX)

S7 Table Interaction effects for personal development (corresponds to Fig 4A). (DOCX)

S8 Table Interaction effects for professional development (corresponds to Fig 4B). (DOCX)

S9 Table Full Poisson regression model for personal development. (DOCX)

S10 Table Full Poisson regression model for professional development. (DOCX)
