# Supplementary material for: Gender discrimination and personal and professional development fostered by allopathic medical schools in the United States
Source: PLoS One. 2026 Jun 22;21(6):e0319549. doi: 10.1371/journal.pone.0319549 (PMC13286186; doi:10.1371/journal.pone.0319549)
Supplement: S1 Table — (DOCX) [file pone.0319549.s001.docx]

# S1 Table. Female students: Frequency of gender discrimination (corresponds to Figure 1)

| Category | N | Percent |
| --- | --- | --- |
| Never | 12,249 | 67.3% |
| Isolated | 2,293 | 12.6% |
| Recurrent | 3,658 | 20.1% |
