# Supplementary material for: Gender discrimination and personal and professional development fostered by allopathic medical schools in the United States
Source: PLoS One. 2026 Jun 22;21(6):e0319549. doi: 10.1371/journal.pone.0319549 (PMC13286186; doi:10.1371/journal.pone.0319549)
Supplement: S2 Table — (DOCX) [file pone.0319549.s002.docx]

**S2 Table. Male students: Frequency of gender discrimination (corresponds to Figure 1)**

| Category | N | Percent |
| --- | --- | --- |
| Never | 17,372 | 89.5% |
| Isolated | 835 | 4.3% |
| Recurrent | 1,203 | 6.2% |
