# Supplementary material for: Gender discrimination and personal and professional development fostered by allopathic medical schools in the United States
Source: PLoS One. 2026 Jun 22;21(6):e0319549. doi: 10.1371/journal.pone.0319549 (PMC13286186; doi:10.1371/journal.pone.0319549)
Supplement: S3 Table — (DOCX) [file pone.0319549.s003.docx]

**S3 Table. Personal development by sex (corresponds to Figure 2A)**

| Sex | N | % Personal Dev | aRR | 95% CI (lower-upper) |
| --- | --- | --- | --- | --- |
| Male | 14,246 | 73.4% | Reference |  |
| Female | 12,950 | 71.2% | 0.97 | 0.96–0.98 |
