# Supplementary material for: Gender discrimination and personal and professional development fostered by allopathic medical schools in the United States
Source: PLoS One. 2026 Jun 22;21(6):e0319549. doi: 10.1371/journal.pone.0319549 (PMC13286186; doi:10.1371/journal.pone.0319549)
Supplement: S4 Table — (DOCX) [file pone.0319549.s004.docx]

# **S4 Table. Professional development by sex (corresponds to Figure 2B)**

| Sex | N | % Professional Dev | aRR | 95% CI (lower-upper) |
| --- | --- | --- | --- | --- |
| Male | 16,796 | 91.2% | Reference |  |
| Female | 17,705 | 92.2% | 1.01 | 1.00–1.02 |
