# Supplementary material for: Gender discrimination and personal and professional development fostered by allopathic medical schools in the United States
Source: PLoS One. 2026 Jun 22;21(6):e0319549. doi: 10.1371/journal.pone.0319549 (PMC13286186; doi:10.1371/journal.pone.0319549)
Supplement: S5 Table — (DOCX) [file pone.0319549.s005.docx]

**S5 Table. Female PPIF by discrimination frequency (corresponds to Figure 3A and 3B)**

| Level | N (Personal) | % Personal | aRR | 95% CI | N (Prof) | % Professional | aRR | 95% CI (lower-upper) |
| --- | --- | --- | --- | --- | --- | --- | --- | --- |
| None | 9,443 | 77.2% | Reference |  | 11,565 | 94.5% | Reference |  |
| Isolated | 1,549 | 67.3% | 0.87 | 0.84–0.90 | 2,138 | 92.8% | 0.98 | 0.96–0.99 |
| Recurrent | 1,957 | 53.3% | 0.69 | 0.67–0.71 | 3,094 | 84.2% | 0.89 | 0.87–0.90 |
