# Supplementary material for: Gender discrimination and personal and professional development fostered by allopathic medical schools in the United States
Source: PLoS One. 2026 Jun 22;21(6):e0319549. doi: 10.1371/journal.pone.0319549 (PMC13286186; doi:10.1371/journal.pone.0319549)
Supplement: S6 Table — (DOCX) [file pone.0319549.s006.docx]

**S6 Table. Male PPIF by discrimination frequency (corresponds to Figure 3C and 3D)**

| Level | N (Personal) | % Personal | aRR | 95% CI | N (Prof) | % Professional | aRR | 95% CI (lower-upper) |
| --- | --- | --- | --- | --- | --- | --- | --- | --- |
| None | 13,185 | 75.9% | Reference |  | 16,145 | 92.9% | Reference |  |
| Isolated | 504 | 59.9% | 0.79 | 0.74–0.84 | 703 | 84.0% | 0.90 | 0.87–0.93 |
| Recurrent | 557 | 46.5% | 0.61 | 0.58–0.66 | 857 | 71.9% | 0.78 | 0.74–0.81 |
