# Supplementary material for: Gender discrimination and personal and professional development fostered by allopathic medical schools in the United States
Source: PLoS One. 2026 Jun 22;21(6):e0319549. doi: 10.1371/journal.pone.0319549 (PMC13286186; doi:10.1371/journal.pone.0319549)
Supplement: S7 Table — (DOCX) [file pone.0319549.s007.docx]

**S7 Table. Interaction effects for personal development (corresponds to Figure 4A)**

| Sex | Contrast | Δ % | aRR | 95% CI (lower-upper) | p-value |
| --- | --- | --- | --- | --- | --- |
| Female | None→Isolated | 13.0% | — | — | — |
| Female | Isolated→Recurrent | 20.9% | — | — | — |
| Male | None→Isolated | 21.1% | 0.90 | 0.88–0.92 | <0.001 |
| Male | Isolated→Recurrent | 21.7% | — | — | — |
