# Supplementary material for: Gender discrimination and personal and professional development fostered by allopathic medical schools in the United States
Source: PLoS One. 2026 Jun 22;21(6):e0319549. doi: 10.1371/journal.pone.0319549 (PMC13286186; doi:10.1371/journal.pone.0319549)
Supplement: S8 Table — (DOCX) [file pone.0319549.s008.docx]

**S8 Table. Interaction effects for professional development (corresponds to Figure 4B)**

| Sex | Contrast | Δ % | aRR | 95% CI (lower-upper) | p-value |
| --- | --- | --- | --- | --- | --- |
| Female | None→Isolated | 2.1% | — | — | — |
| Female | Isolated→Recurrent | 9.7% | — | — | — |
| Male | None→Isolated | 9.7% | 0.93 | 0.92–0.94 | <0.001 |
| Male | Isolated→Recurrent | 14.3% | 0.95 | 0.93–0.97 | <0.001 |
