# Supplementary material for: Gender discrimination and personal and professional development fostered by allopathic medical schools in the United States
Source: PLoS One. 2026 Jun 22;21(6):e0319549. doi: 10.1371/journal.pone.0319549 (PMC13286186; doi:10.1371/journal.pone.0319549)
Supplement: S9 Table — (DOCX) [file pone.0319549.s009.docx]

**S9 Table. Full Poisson regression model for personal development**

| Variable | aRR | Std Err | z | P>\|z\| | 95% CI (lower-upper) |
| --- | --- | --- | --- | --- | --- |
| sex_num: F | 1.01 | 0.002 | 6.49 | <0.001 | 1.01-1.02 |
| exp_discrim:Isolated | 0.78 | 0.007 | -27.32 | <0.001 | 0.76-0.79 |
| exp_discrim:Recurrent | 0.58 | 0.006 | -52.66 | <0.001 | 0.57-0.59 |
| F#Isolated | 1.11 | 0.011 | 9.80 | <0.001 | 1.09-1.13 |
| F#Recurrent | 1.14 | 0.014 | 11.05 | <0.001 | 1.11-1.17 |
| Asian | 1.01 | 0.003 | 3.18 | 0.001 | 1.00-1.01 |
| Black | 0.87 | 0.005 | -26.93 | <0.001 | 0.87-0.88 |
| Hispanic | 1.01 | 0.004 | 2.76 | 0.006 | 1.00-1.02 |
| Multiracial | 1.02 | 0.005 | 4.95 | <0.001 | 1.01-1.03 |
| Other | 0.95 | 0.006 | -8.64 | <0.001 | 0.93-0.96 |
| <50k | 0.98 | 0.003 | -8.30 | <0.001 | 0.96-0.98 |
| <75k | 0.99 | 0.004 | -3.24 | 0.001 | 0.99-1.00 |
| <125k | 1.00 | 0.003 | 1.04 | 0.300 | 1.00-1.01 |
| Variable | aRR | Std Err | z | P>\|z\| | 95% CI (lower-upper) |
| <200k | 1.01 | 0.004 | 2.64 | 0.008 | 1.00-1.02 |
| >=200k | 1.00 | - | - | - | - |
| Constant | 0.76 | 0.002 | -91.99 | <0.001 | 0.76-0.77 |
