# Supplementary material for: Gender discrimination and personal and professional development fostered by allopathic medical schools in the United States
Source: PLoS One. 2026 Jun 22;21(6):e0319549. doi: 10.1371/journal.pone.0319549 (PMC13286186; doi:10.1371/journal.pone.0319549)
Supplement: S10 Table — (DOCX) [file pone.0319549.s010.docx]

**S10 Table. Full Poisson regression model for professional development**

| Variable | aRR | Std Err | z | P>\|z\| | 95% CI (lower-upper) |
| --- | --- | --- | --- | --- | --- |
| sex_num: F | 1.03 | 0.002 | 23.90 | <0.001 | 1.02-1.03 |
| exp_discrim:Isolated | 0.96 | 0.004 | -20.27 | <0.001 | 0.90-0.91 |
| exp_discrim:Recurrent | 0.77 | 0.004 | -44.38 | <0.001 | 0.77-0.78 |
| F#Isolated | 1.07 | 0.006 | 13.24 | <0.001 | 1.06-1.09 |
| F#Recurrent | 1.13 | 0.007 | 19.20 | <0.001 | 1.12-1.14 |
| Asian | 0.99 | 0.001 | -11.48 | <0.001 | 0.98-0.99 |
| Black | 0.94 | 0.002 | -23.49 | <0.001 | 0.94-0.95 |
| Hispanic | 0.94 | 0.003 | -27.00 | <0.001 | 0.94-0.99 |
| Multiracial | 0.99 | 0.004 | -4.01 | <0.001 | 0.98-0.99 |
| Other | 0.96 | 0.003 | -11.35 | <0.001 | 0.96-0.97 |
| <50k | 0.99 | 0.001 | -8.31 | <0.001 | 0.98-0.99 |
| <75k | 1.00 | 0.002 | 0.53 | 0.594 | 1.00-1.01 |
| Variable | aRR | Std Err | z | P>\|z\| | 95% CI (lower-upper) |
| <125k | 1.01 | 0.002 | 7.45 | <0.001 | 1.01-1.02 |
| <200k | 1.02 | 0.002 | 8.44 | <0.001 | 1.01-1.02 |
| >=200k | 1.00 | - | - | - | - |
| Constant | 0.93 | 0.001 | -50.52 | <0.001 | 0.92-0.93 |
