# Supplementary material for: Gender discrimination and personal and professional development fostered by allopathic medical schools in the United States
Source: PLoS One. 2026 Jun 22;21(6):e0319549. doi: 10.1371/journal.pone.0319549 (PMC13286186; doi:10.1371/journal.pone.0319549)
Supplement: Attachment — Submitted filename: Binary Gender Classification.docx [file pone.0319549.s012.docx]

1. **Binary Gender Classification**
   The study excludes non-binary individuals, limiting the scope of analysis despite addressing "gender discrimination."
2. **Weak Operationalization of PPIF**
   Personal and professional identity formation is measured via unvalidated single-item indicators, insufficient for such a complex construct.
3. **Unclear Discrimination Categories**
   The classification of gender discrimination lacks methodological transparency and may not reflect actual experiential differences.
4. **Insufficient Interaction Modeling**
   Analyses of gender × discrimination interaction effects are inadequately described and potentially underadjusted for confounders.
5. **High Missing Data and Imputation Dependence**
   Heavy reliance on imputed data (up to 27% missingness) without detailed diagnostics weakens the credibility of findings.
6. **Interpretation Risk: Male Vulnerability**
   Claims that men experience sharper declines in PPIF may reflect perception/reporting biases rather than true differential impact.
7. **Lack of Qualitative Context**
   Absence of narrative or qualitative data limits depth and interpretability of the results in such a nuanced area.
8. **Generic Recommendations**
   Conclusions lack specific, actionable strategies for institutions; practical implications are underdeveloped.
