# Supplementary material for: Gender discrimination and personal and professional development fostered by allopathic medical schools in the United States
Source: PLoS One. 2026 Jun 22;21(6):e0319549. doi: 10.1371/journal.pone.0319549 (PMC13286186; doi:10.1371/journal.pone.0319549)
Supplement: Attachment — Submitted filename: Response to Reviewers.docx [file pone.0319549.s013.docx]

|  | **Department of Emergency Medicine** |
| --- | --- |
|  | Translational Research Building |
|  | 227 East 30th Street, 1st floor, NY, 10016 |
|  | T 475-241-5322  E [shruthi.venkataraman@nyulangone.org](mailto:shruthi.venkataraman@nyulangone.org) |
|  |  |

August 10, 2025

Somayeh Delavari, Ph.D.,
Academic Editor
PLOS ONE

Dear Dr. Delavari,

Thank you for you review of our manuscript, “Gender Discrimination and Medical Student Development.” We appreciate the reviewers’ thoughtful comments and recommendations and have responded to each concern and suggestion below:

**JOURNAL REQUIREMENTS:**

1. When submitting your revision, we need you to address these additional requirements.

Please ensure that your manuscript meets PLOS ONE's style requirements, including those for file naming. The PLOS ONE style templates can be found at

<https://journals.plos.org/plosone/s/file?id=wjVg/PLOSOne_formatting_sample_main_body.pdf> and <https://journals.plos.org/plosone/s/file?id=ba62/PLOSOne_formatting_sample_title_authors_affiliations.pdf>

**Response:** We have now made modifications to bring the manuscript to compliance with PLOS One’s style requirements.

2. Thank you for stating the following financial disclosure:

Alexis Webber, Mytien Nguyen, Hyacinth RC Mason, Mayur M. Desai, and Shruthi Venkataraman report no conflicts of interest. Dowin Boatright received NIH grant (R35GM153263) support. Alexandra Hajduk received NIH grants (R01 GM146147, R01 MD018928, R01 HL160822) and Burroughs-Welcome Fund travel support. Mytien received NIGMS (T32GM136651) and NIAID (F30AI157227) grant support. Sarwat Chaudhry received support from NIH/DHHS (1R01MD018928-01A1) and NIH/NIGMS (GM146147). Tonya L. Fancher received grants and consulting fees from the American Medical Association, honoraria from Montefiore School of Medicine, Yale School of Medicine, University of Minnesota School of Medicine, travel support from ACGME, and holds a leadership role with HRSA.

Please state what role the funders took in the study.  If the funders had no role, please state: "The funders had no role in study design, data collection and analysis, decision to publish, or preparation of the manuscript."

If this statement is not correct you must amend it as needed.

Please include this amended Role of Funder statement in your cover letter; we will change the online submission form on your behalf.

**Response:** We have now amended the Role of Funder statement in our cover letter.

**Modified Role of Funder statement in cover letter:** “The funders had no role in study design, data collection and analysis, decision to publish, or preparation of the manuscript.”

3. Thank you for stating the following in the Acknowledgments Section of your manuscript:

This work is supported by the National Institute of Health: NIGMS grant T32GM136651 (MN), NIAID grant F30AI157227 (MN), NIGMS grant R35GM153263 (DB). The funder had no role in the design and conduct of the study; collection, management, analysis, and interpretation of the data; preparation, review, or approval of the manuscript; and decision to submit the manuscript for publication. This material is based upon data provided by the Association of American Medical Colleges (“AAMC”). The views expressed herein are those of the authors and do not necessarily reflect the position or policy of the AAMC.

We note that you have provided funding information that is not currently declared in your Funding Statement. However, funding information should not appear in the Acknowledgments section or other areas of your manuscript. We will only publish funding information present in the Funding Statement section of the online submission form. Please remove any funding-related text from the manuscript and let us know how you would like to update your Funding Statement. Currently, your Funding Statement reads as follows: Alexis Webber, Mytien Nguyen, Hyacinth RC Mason, Mayur M. Desai, and Shruthi Venkataraman report no conflicts of interest. Dowin Boatright received NIH grant (R35GM153263) support. Alexandra Hajduk received NIH grants (R01 GM146147, R01 MD018928, R01 HL160822) and Burroughs-Welcome Fund travel support. Mytien received NIGMS (T32GM136651) and NIAID (F30AI157227) grant support. Sarwat Chaudhry received support from NIH/DHHS (1R01MD018928-01A1) and NIH/NIGMS (GM146147). Tonya L. Fancher received grants and consulting fees from the American Medical Association, honoraria from Montefiore School of Medicine, Yale School of Medicine, University of Minnesota School of Medicine, travel support from ACGME, and holds a leadership role with HRSA.

Please include your amended statements within your cover letter; we will change the online submission form on your behalf.

**Response:** We have now removed all funding-related text from the manuscript and have included an accurate Funding Statement within our cover letter, for transfer to the online submission form by PLOS One.

**Modified Funding Statement included in the cover letter:** “Authors Shruthi Venkataraman, Alexis Webber, Mytien Nguyen, Hyacinth RC Mason, and Mayur M. Desai report no conflicts of interest. Dowin Boatright received NIH grant (R35GM153263) support. Alexandra Hajduk received NIH grants (R01 GM146147, R01 MD018928, R01 HL160822) and Burroughs-Welcome Fund travel support. Mytien Nguyen received NIGMS (T32GM136651) and NIAID (F30AI157227) grant support. Sarwat Chaudhry received support from NIH/DHHS (1R01MD018928-01A1) and NIH/NIGMS (GM146147). Tonya L. Fancher received grants and consulting fees from the American Medical Association, honoraria from Montefiore School of Medicine, Yale School of Medicine, University of Minnesota School of Medicine, travel support from ACGME, and holds a leadership role with HRSA. The funders had no role in study design, data collection and analysis, decision to publish, or preparation of the manuscript. The views expressed in the manuscript are those of the authors and do not necessarily reflect the position or policy of the AAMC.”

4. In the online submission form, you indicated that your data is available only on request from a third party. Please note that your Data Availability Statement is currently missing the contact details for the third party, such as an email address or a link to where data requests can be made. Please update your statement with the missing information.

**Response:** We have now added a Data Availability Statement with a link to where data requests from the AAMC can be made and an email address to which queries regarding data requests can be addressed.

**Modified Data Availability Statement in the online submission form:** “The data underlying the results presented in this study are available upon request from the Association of American Medical Colleges (AAMC). Requests can be submitted via the online form at <https://www.aamc.org/request-aamc-data>. Questions regarding data access may be directed to datarequest@aamc.org.”

**REVIEWER’S COMMENTS**

**Reviewer #1:** 

1. The current study is a valuable study on gender discrimination. However, the following questions need further investigation:

   Is this study applicable to other societies and cultures? This needs to be addressed in the discussion section.

**Response:** We thank Reviewer 1 for raising an important point regarding global relevance of study findings. Our study utilizes data collected in the United States. As such, study implications may or may not have relevance to other medical schools outside the US. However, the issue of gender discrimination in medical education eclipses the Global North and there is evidence of gender discrimination in medical education in countries in the Global South, though its manifestations and cultural underpinnings may vary (Newman, 2016; Wong, 2009; Hashmi, 2013). For instance, a large study conducted among medical students in Brazil suggest a link between gender discrimination and factors influencing professional development, such as burnout, wellbeing, and self-confidence (Gerk, 2022). Further, a qualitative study of female physicians at different stages their career in Japan found that the process of professional identity formation was affected by gender stereotypes (Matsui, 2019). We have now added a paragraph to the Discussion on the global relevance of our findings, citing evidence from other contexts that suggests applicability of our findings and noting that while our findings may resonate with patterns observed internationally, more comparative research is needed to understand differences in the prevalence, quality and consequences of gender discrimination in medical education in other contexts.

References:

1) Newman, C., Ng, C., Pacqué-Margolis, S., & Frymus, D. (2016). Integration of gender-transformative interventions into health professional education reform for the 21st century: implications of an expert review. *Human resources for health*, *14*, 1-12.
2) Wong, Y. L. (2009). Gender competencies in the medical curriculum: addressing gender bias in medicine. *Asia Pacific Journal of Public Health*, *21*(4), 359-376.
3) Hashmi, A. M., Rehman, A., Butt, Z., Aftab, M. A., Shahid, A., & Khan, S. A. (2013). Gender discrimination among medical students in Pakistan: a cross sectional survey. *Pakistan Journal of Medical Sciences*, *29*(2), 449.
4) Gerk, A., Campos, L., Naus, A., Faria, I., Buda, A. M., Moura, C. B., ... & Ferreira, J. L. (2022). Gender discrimination, career aspirations, and access to mentorship among medical students in Brazil. *Journal of Surgical Research*, *279*, 702-711.
5) Matsui, T., Sato, M., Kato, Y., & Nishigori, H. (2019). Professional identity formation of female doctors in Japan–gap between the married and unmarried. *BMC medical education*, *19*, 1-9.

**Modified text (Discussion; lines 447-461):** “Applicability of study findings to other contexts

Our study draws on data from U.S. medical schools and may or may not be generalizable to medical educational settings outside North America where sociocultural norms, gender roles, and institutional structures can differ and differentially influence the experience and impact of gender discrimination. However, there is evidence that gender discrimination also influences PPIF among medical students in other national contexts. In Brazil, a large survey of medical students found that experiences of gender discrimination among cisgender women and nonbinary individuals were associated with lower career satisfaction, diminished self-confidence, increased burnout, and limited access to mentorship, all of which can influence professional identity formation. [39] In Japan, a qualitative study of female physicians reflecting on their training experiences found that gender stereotyping disrupted the integration of personal and professional identities, complicating the development of a stable professional identity.[40] These studies support the broader relevance of our findings and underscore the need for more cross-national research to examine how these dynamics unfold in diverse educational and cultural environments.”

2. The authors used existing data collected over time. It seems that a supplementary study in the form of interviews (qualitative study) is needed to support the data.

**Response:** We thank Reviewer 1 for pointing out the importance of integrating qualitative data to further contextualize experiences of gender discrimination in medical education and how it may shape identity formation. We would like to clarify that the large-scale data we had access to was purely quantitative. As such, we have now integrated findings from existing qualitative work exploring how gender discrimination in medical education may influence professional identity formation among women in the ‘Discussion’ section. We were unable to identify qualitative studies that contextualize our findings among men (and noted this in the ‘Discussion’) and found a paucity of longitudinal qualitative research on this topic; we have therefore included these as future directions in the ‘Conclusion’ section.

**Modified text (Discussion; lines 400-412):** “Qualitative research suggests that gender discrimination in medical school shapes professional identity formation among women by restricting access to meaningful participation, recognition, and support in clinical training environments.[3,33,34] Across studies, women reported being perceived as less capable, subjected to higher performance thresholds, or overlooked for key learning opportunities, which disrupted their sense of belonging, narrowed their imagined futures in medicine, and in some cases influenced specialty choice.[3,33,34] Some described feeling unequipped to respond to inappropriate behavior from male supervisors, leading to emotional withdrawal, self-blame, and normalization of these experiences as part of becoming a physician.[3,9] The absence of relatable role models and fear of speaking up further limited how women could safely reflect on or challenge these experiences.[9,33,34] These processes were reinforced by gendered double standards, where women were penalized both for lacking confidence and for displaying it, revealing how professional legitimacy was tethered to conflicting and exclusionary norms.[3,33]”

**Modified text (Discussion; lines 426-433):** “However, little qualitative research has examined how men, particularly men of color, experience gender discrimination in medical education, despite the possibility that their experiences may be shaped by intersecting racial and gendered dynamics that differ from those of white men or women.[37] This gap limits our ability to fully interpret the stronger declines in PPIF observed among men, particularly in understanding how men perceive, experience, and respond to gender discrimination, and highlights the need for intersectional qualitative research that captures the experiences of men, women, and gender-diverse students across intersectional identities.

**Modified text (Conclusion; lines 524-527):** “Future research is needed to understand how gender discrimination influences the holistic development of medical students across the spectrum of gender identity and expression, using longitudinal qualitative and mixed methods approaches that include women, men, and gender-diverse students with intersecting marginalized identities.”

3. What do the authors suggest for improving the situation? What has been stated in similar studies?

**Response:** In the ‘Implications’ section, we had proposed several strategies to mitigate gender discrimination in medical education, namely: 1) integrating personal and professional identity formation as critical equity metrics and formally mandating parity for students across all racial and ethnic (based on our prior work), and gender backgrounds in the LCME accreditation standards, 2) for educators, institutions, and organizations like the LCME to consider implementing initiatives to combat gender bias, which would benefit not only students who are women but also enhance the development of students who are men, who may experience more significant declines in personal and professional identity formation with increased gender discrimination, 4) for institutions to foster a sense of belonging and promote professional development for learners experiencing marginalization in medicine by transforming academic medicine environments to recognize, hear, and value their contributions, 5) utilizing validated tools, such as the PRODIGIE tool developed by our team, to systematically measure the climate of equity and inclusion within the medical school learning environment, which can help identify actionable targets to improve the climate, thus improving educational outcomes for all students. We had cited prior studies on the topic that support these recommendations, as appropriate. We have now reframed this section as the ‘Implications and Recommendations’ section and included other strategies suggested by studies on the topic.

**Modified text (Implications and Recommendations; lines 490-512):** “Our findings highlight a persisting need for systemic reforms in medical education to address gender discrimination that is experienced by both female and male students, and to further investigate how gender discrimination may influence educational outcomes among gender-diverse students nationally. Addressing gender discrimination in the learning environment, as well as racial discrimination, as our previous work suggests, may serve to promote holistic development for all students.[42] We propose considering the integration of PPIF as a critical equity metric and formally including PPIF parity for students of all racial, ethnic, and gender backgrounds in the LCME accreditation standards. Educators, institutions, and organizations like the LCME should consider implementing initiatives to combat gender bias, such as implicit bias training for hiring committees, faculty, and students; framing these as benefiting not only students who are women but also enhancing the development of students who are men, who may experience more significant declines in PPIF with increased gender discrimination. Fostering a sense of belonging for minoritized and marginalized learners in medicine—by uncovering unconscious biases and transforming academic medicine environments to recognize, hear, and value their contributions—can positively influence PPIF in medical school.[43,44] Validated tools that can systematically measure and help achieve a climate of equity and inclusion within the medical school learning environment, such as the PRODIGIE tool developed by our team, may provide medical schools with actionable insights to foster equity and inclusion,[45] ultimately improving educational outcomes, including PPIF, for all students. Additional strategies to mitigate gender discrimination in medical education include increasing the representation of women and gender-diverse individuals in academic medicine leadership,[46,47] and developing formal anti-harassment policies that clearly define unacceptable behaviors.[48]

”

4. The statement of the problem is not well explained. This needs to be addressed different articles and their differing views on this matter. There is a need to further explain the context of the study and also to explain the differences between the contexts on this issue.

**Response:** We appreciate this important comment and have undertaken a full revision of the ‘Introduction’ section to directly address it. We have now clearly defined the central problem: the underexplored relationship between gender discrimination and medical students’ personal and professional identity formation. In contrast to the original version, which mentioned this gap briefly at the end, the revised version centers it. To address the request for engagement with differing views and broader context, we incorporated findings from studies in the U.S., Germany, the UK, and Scandinavia. These illustrate variation in how gender discrimination is experienced and interpreted across settings—for example, differing impacts on specialty choice, varied gender distributions in reported experiences, and the role of implicit bias and symbolic messaging. We also expanded the description of how gender discrimination manifests—from overt harassment to hidden curriculum dynamics—and distinguished gender-specific experiences, such as male students’ marginalization in female-dominated fields and female students’ behavioral adaptation to fit norms. Finally, we clarified why our national quantitative study is needed by noting that most prior work in this area has been qualitative and limited in scope. These changes collectively aim to clarify the problem, justify the study, and situate it within the literature.

**Modified text (Introduction; lines 84-146):** “Gender-based bias and discrimination remain pervasive in medical training, with far-reaching implications for medical students’ development. Studies conducted in different contexts underscore that this problem is widespread yet manifests in varied ways. For example, a 14-school survey in the United States found that 93% of female and 83% of male students had encountered at least one incident of gender discrimination or harassment during medical school.[1] Nearly half of those women reported that these negative experiences influenced their choice of specialty, compared to 16% of affected men.[1] Similarly, a recent survey in Germany documented frequent gender bias: 88% of women reported experiencing gender discrimination, versus 46% of men.[2] Notably, one in ten female respondents in that study said they avoided or ruled out certain specialties entirely due to anticipated gender-based bias, a striking indication of how deeply these perceptions can shape career trajectories.[2] In a 1995 report, the Council on Graduate Medical Education in the United States stated: “Gender bias, a reflection of society's value system, remains the single greatest deterrent to women achieving their full potential in every aspect of the medical profession and is a barrier throughout the professional life cycle.”[1]

Importantly, gender discrimination in medical school is not always overt; it often operates through subtle “hidden curriculum” signals. Qualitative research in the UK and Scandinavia describes how everyday interactions – from gender‐stereotyped expectations and offhand remarks to the dearth of female role models in certain fields – convey implicit messages about who belongs where.[2,3] Female students report having to work harder to “fit in,” sometimes altering their behavior or appearance to navigate gendered expectations.[4] At the same time, context matters. In traditionally female-dominated fields like obstetrics and gynecology, male medical students have reported feeling marginalized because of their gender.[1,4] Even as blatant sexism and harassment have become less socially acceptable over time, research suggests that implicit bias and structural inequities persist. Physical symbols of institutional culture (such as walls filled with portraits of male leaders) and lingering pay and promotion gaps send a message that medicine is still “too male, too pale… too stale,” as one UK study put it.[3] In short, despite progress, gender inequality remains woven into the fabric of medical education across diverse contexts, reinforcing the need to understand its impact on learners.

One critical gap in the literature – and the focus of our study – is how experiences of gender discrimination influence medical students’ personal and professional identity formation (PPIF). Professional identity formation is the process by which trainees internalize the values, behaviors, and sense of belonging to the medical profession. The Association of American Medical Colleges (AAMC) emphasizes the importance of personal and professional development—cultivating qualities necessary for lifelong growth as a person and a physician—as foundational competencies in medical training.[5] The learning environment in medical school shapes identity formation and is influenced by interactions with peers, patients, and supervisors.[6–8]

While the existence of gender discrimination in medical school has been documented, the “full spectrum” of gender-based experiences and its specific effect on students’ emerging identities as physicians is underexplored.[9] Emerging evidence, however, suggests the effect is significant. Babaria et al. observed that third-year female students become acculturated to unprofessional behavior from male supervisors: encountering frequent sexism, they described feelings of guilt and diminished self-confidence but, with time, grew “used to it” and resigned to the idea that such mistreatment was simply part of being a woman doctor.[9] Similarly, a focus-group study from Sweden – a country known for its commitment to gender equality – found that both female and male students adjusted their behavior to cope with a gendered clinical “climate.”[4] Students reported staying silent or avoiding challenging unprofessional behavior from supervisors due to fear of repercussions, indicating how power dynamics and sexism pushed them to conform rather than speak up.[4] Such informal lessons in medical school (the hidden curriculum) have a powerful influence on socialization and identity formation.[10] They can subtly shape how future physicians see themselves and their role in the profession, for example by reinforcing doubts about belonging or steering individuals away from certain identities (e.g. surgeon, leader) that seem “off-limits” to their gender.[3]

Prior studies examining gender bias in medical education have largely been qualitative (offering rich narratives but limited generalizability) and conducted in a select number of institutions. The context of the present study helps address these gaps by examining the influence of graded gender discrimination experience on medical schools’ ability to foster personal and professional identity formation among male and female medical students in the United States using national quantitative data. This work will shed light on an under-addressed aspect of medical training (the influence of gender discrimination on identity formation), thereby helping educators and policy makers better understand how to foster an inclusive learning environment that supports all students’ holistic growth.”

5. The study tools and how to ensure its validity and reliability should be stated.

**Response:** We outline in the ‘Methods’ section that the measures for sex, gender discrimination experience and personal and professional identity formation utilized in this study were obtained from the AAMC data warehouse, specifically the American Medical College Application Service (AMCAS) survey and Graduation Questionnaire (GQ), administered before commencing medical school and at graduation, respectively. We devote paragraphs in the ‘Methods’ section (lines 165-213) to state how these variables were asked on the survey and what the available answer choices were. We also discuss the validity and reliability of these measures.

**Modified text (lines 182-214):** “Experience of Gender Discrimination

Gender discrimination frequency was determined via the following three questions in the GQ: 1) “During medical school, how frequently have you been denied opportunities for training or rewards based on gender?” 2) “During medical school, how frequently have you been subjected to sexist remarks/names?” and 3) “During medical school, how frequently have you received lower evaluations or grades solely because of gender rather than performance?” To each question, participants could respond ‘Never’, ‘Once’, ‘Occasionally’ or ‘Frequently’. We recategorized the variable into three levels: none, isolated, and recurrent. Participants who reported 'None' to all three questions were categorized as having no experiences of gender discrimination. Those who responded ‘Once’ to only one of the questions were categorized as having isolated experiences. Participants were classified as having recurrent experiences if they met one of the following criteria: at least one response of 'Occasionally' or 'Frequently' to any question, or 'Once' to more than one question. These three items are regularly included in the annual AAMC Graduation Questionnaire and are the most widely available national-level indicators of gender-based mistreatment in U.S. medical education. While not part of a validated scale, they exhibit strong face validity as direct, interpretable indicators of serious forms of gender discrimination in medical school settings. To our knowledge, no formal psychometric validation (e.g., internal consistency, factor analysis) of these items has been published.

Outcomes

PPIF was assessed using two distinct metrics: personal and professional development. Personal development was measured by the GQ item “My medical school has done a good job of fostering and nurturing my development as a person” and professional development was measured by the GQ item “My medical school has done a good job of fostering and nurturing my development as a physician”. Responses were recorded on a 5-point Likert scale from ‘Strongly disagree’ to ‘Strongly agree’. Variables were dichotomized such that only participants who expressed strong agreement (i.e., ‘Agree’ or ‘Strongly agree’) were considered as reporting that their medical school nurtured their PPIF. Both personal and professional development were examined separately in all analyses; the term PPIF is used to collectively refer to these two outcomes. The two outcome items originated from a single-institution study at Wake Forest University in 1996 [11] and were subsequently adopted into the AAMC GQ. While they are not components of a validated scale and cannot be tested for reliability as single items, they are used in national research and institutional evaluation as global indicators of student perceptions of support for identity formation.[12,13]”

**Reviewer #2:**

1. Binary Gender Classification

The study excludes non-binary individuals, limiting the scope of analysis despite addressing "gender discrimination."

**Response:** We thank Reviewer 2 for raising this important point. Currently, the AAMC does not release data on non-binary individuals due to the small number and concerns about anonymity. We would also like to clarify that while we initially referred to the variable as “gender,” but we have since received clarification from the AAMC that the AMCAS application used the label “Sex,” with response options of “Male,” “Female,” and “Decline to Answer.” The item did not specify whether it referred to sex assigned at birth. Thus, we acknowledge that our binary variable likely captures a mixture of sex and gender identity, and we have revised the manuscript throughout to use “sex” when referring to this variable. We also now clearly note this limitation in the ‘Methods’ and ‘Limitations’ sections. Furthermore, we have added language to the Conclusion emphasizing the need for future research examining the experiences of medical students across the spectrum of gender identity and expression.

**Modified text (Methods; lines 167-174):** “Data on sex, race and ethnicity, and childhood household income were obtained from the AMCAS. Students self-reported their sex as male, female, or ‘decline to answer’. The item was labeled ‘Sex’ on the 2014 and 2015 AMCAS application and was not accompanied by a definition or clarification about whether it referred to sex assigned at birth. As such, this variable may reflect sex, gender identity, or a combination thereof, depending on how applicants interpreted the question. For analytic clarity, we refer to participants as 'male' or 'female' based on their selection in response to this item, while acknowledging this limitation in our interpretation.”

**Modified text (Limitations; lines 463-470):** “Our binary sex variable (female/male) does not capture intersex variations and should not be conflated with gender identity. For our study years, the AAMC did not release data on intersex and non-binary individuals because of the small sample size and concerns about maintaining anonymity. Although we refer to this variable as ‘sex,’ the AMCAS item lacked a definition, which may have led some respondents to interpret it as gender identity, introducing potential misclassification. Prior work suggest that, in the absence of clarification, respondents may conflate sex and gender when completing survey items.[41] This ambiguity underscores the need for inclusive, clearly defined measures in future research.”

**Modified text (Conclusion; lines 524-527):** “Future research is needed to understand how gender discrimination influences the holistic development of medical students across the spectrum of gender identity and expression, using longitudinal qualitative and mixed methods approaches that include women, men, and gender-diverse students with intersecting marginalized identities.

2. Weak Operationalization of PPIF

Personal and professional identity formation is measured via unvalidated single-item indicators, insufficient for such a complex construct.

**Response:** We agree that personal and professional identity formation likely extends beyond institutional support for students’ personal and professional development and have thus acknowledged it as a limitation. This operationalization has been previously used as a precedent in prior peer-reviewed literature (Nguyen, 2025; Nguyen, 2022; Venkataraman, 2024) and allows us to leverage the AAMC’s vast national data. We now comment on the validity and reliability of these measures.

References:

1) Nguyen, M., Mason, H. R., Fancher, T., Chaudhry, S. I., Tran-Reina, M. L., Havemann, C., ... & Boatright, D. (2025). Medical School Support of Students’ Professional and Personal Development by Sociodemographic Characteristics. *JAMA Network Open*, *8*(5), e2511063-e2511063.
2) Nguyen, M., Mason, H. R., Russell, R. G., & Boatright, D. (2022). Professional identity formation among diverse US medical students: a quantitative analysis of the impact of socioeconomic status. *Academic Medicine*, *97*(11S), S170.
3) Venkataraman, S., Nguyen, M., Chaudhry, S. I., Desai, M. M., Hajduk, A. M., Mason, H. R., ... & Boatright, D. (2024). Racial and Ethnic Discrimination and Medical Students’ Identity Formation. *JAMA Network Open*, *7*(10), e2439727-e2439727.

**Associated text (Methods; lines 209-214):** “The two outcome items originated from a single-institution study at Wake Forest University in 1996 [11] and were subsequently adopted into the AAMC GQ. While they are not components of a validated scale and cannot be tested for reliability as single items, they are used in national research and institutional evaluation as global indicators of student perceptions of support for identity formation. [12,13]”

**Associated text (Limitations; lines 480-486):** “While the GQ measures used as proxies for personal and professional development assess institutional success in fostering student growth—perhaps appropriately attributing responsibility to the school—they may not fully capture the extent of development, or lack thereof, experienced by students. We recognize that the multi-domain and dynamic nature of identity development during medical training likely extends beyond the development fostered by the institution to also include individual factors, though institutional efforts remain crucial.[6,8]”

3. Unclear Discrimination Categories
   The classification of gender discrimination lacks methodological transparency and may not reflect actual experiential differences.

**Response:** We report in the methods how gender discrimination was assessed and operationalized. Self-reported gender discrimination was assessed based on the frequency of students’ experiences of: 1) being denied opportunities for training or rewards based on gender, 2) being subjected to sexist remarks/names, 3) receiving lower evaluations or grades solely because of gender rather than performance. These items have collectively assessed concrete instances of serious gender discrimination that have the capacity to inflict multifaceted injury on a national survey by the AAMC for the past 13 years. We do, however, agree that these may be only a subset of gender discrimination experiences, as acknowledged in the ‘Limitations’ section.

**Associated text (Methods, lines 183-194):** “Gender discrimination frequency was determined via the following three questions in the GQ: 1) “During medical school, how frequently have you been denied opportunities for training or rewards based on gender?” 2) “During medical school, how frequently have you been subjected to sexist remarks/names?” and 3) “During medical school, how frequently have you received lower evaluations or grades solely because of gender rather than performance?” To each question, participants could respond ‘Never’, ‘Once’, ‘Occasionally’ or ‘Frequently’. We recategorized the variable into three levels: none, isolated, and recurrent. Participants who reported 'None' to all three questions were categorized as having no experiences of gender discrimination. Those who responded ‘Once’ to only one of the questions were categorized as having isolated experiences. Participants were classified as having recurrent experiences if they met one of the following criteria: at least one response of 'Occasionally' or 'Frequently' to any question, or 'Once' to more than one question.”

**Associated text (Limitations, lines 478-480):** “Furthermore, the GQ captures only a subset of gender discrimination experiences; however, those included are significant and can have serious psychological and professional impacts.”

4. Insufficient Interaction Modeling
   Analyses of gender × discrimination interaction effects are inadequately described and potentially underadjusted for confounders.

**Response:** We appreciate the opportunity to clarify. To evaluate sex differences in the association between gender discrimination and PPIF, we used two complementary approaches. First, in Figure 3, we estimated adjusted relative risks for each combination of sex and discrimination level by creating a composite 6-level categorical variable. This approach allowed for clear group-level comparisons (e.g., male with recurrent discrimination vs. female with recurrent discrimination) but did not formally test statistical interaction. In contrast, Figure 4 presents results from a formal interaction model that included a cross-product term between binary sex and 3-level discrimination. To interpret the interaction, we used post-estimation margins to estimate adjusted predicted probabilities for personal and professional development across discrimination levels by sex. We then used linear contrasts to test whether the changes in predicted probability across increasing levels of discrimination differed by sex. These probabilities and differences are visualized using margins plots. We have now clarified these distinctions in the Methods section. Across both analyses, we adjusted for critical confounders such as race and ethnicity and socioeconomic status.

**Modified text (Statistical analysis, lines 234-245):** “To examine sex differences in the association between gender discrimination and PPIF, we used two complementary modeling approaches. First, we created a composite 6-level categorical variable representing all combinations of sex (male/female) and discrimination frequency (never, isolated, recurrent), which we used as the main predictor in Poisson regression models with robust standard errors to estimate adjusted relative risks for each group. Second, to formally test for interaction, we specified a Poisson regression model that included a cross-product term between sex and discrimination frequency. We used post-estimation margins to calculate adjusted predicted probabilities and applied linear contrasts to evaluate whether the magnitude of change in outcome probabilities across increasing discrimination levels differed by sex. Two-sided *p* < .05 indicated significance. All relative risk estimates are adjusted for race, ethnicity and childhood household income.”

”

5. High Missing Data and Imputation Dependence
   Heavy reliance on imputed data (up to 27% missingness) without detailed diagnostics weakens the credibility of findings.

**Response:** We thank the reviewer for this important observation. As noted, several key variables had substantial missingness, with a maximum of 27.5%. We addressed missingness using multiple imputation by chained equations, incorporating all sociodemographic, exposure, and outcome variables into the imputation model. We conducted diagnostic checks including convergence monitoring and comparison of marginal distributions between observed and imputed values. In addition, we conducted complete-case sensitivity analyses, which produced estimates similar in direction and magnitude to those from the imputed models. These findings support the robustness of our results. We now describe these diagnostics and limitations more clearly in both the ‘Statistical Analysis’ and ‘Limitations’ sections.

**Modified text (Statistical analysis, lines 225-230):** “We assessed imputation diagnostics by monitoring model convergence and comparing the distributions of observed and imputed values to ensure plausibility and consistency. Sensitivity analyses restricted to complete cases yielded estimates similar in direction and magnitude to those from the imputed datasets. We present the imputed results to reduce potential bias due to listwise deletion and to retain statistical power.”

**Modified text (Limitations, lines 474-478):** “While multiple imputation slightly shifted the gender distribution from a female to a male majority—which could, in theory, attenuate associations—the imputation model included sex, discrimination, and both outcome variables, enabling it to preserve differential outcome patterns by sex. Complete-case and imputed estimates were otherwise similar in direction and magnitude.”

6. Interpretation Risk: Male Vulnerability
   Claims that men experience sharper declines in PPIF may reflect perception/reporting biases rather than true differential impact.

**Response:** We appreciate this thoughtful comment and agree that gender subgroup differences in self-reported outcomes require cautious interpretation. In the ‘Discussion’ section (lines 414-424), we explicitly consider the possibility that sharper declines in PPIF among men may reflect the novelty and social incongruity of experiencing gender discrimination, which may heighten its perceived impact. We frame this pattern through the lens of stereotype threat, limited coping strategies, and gendered expectations about professional belonging. Our interpretation does not imply greater objective harm for men, but rather highlights how differences in exposure, perception, and social context may shape responses to gender-based mistreatment. We also emphasize that these interpretations remain hypothesis-generating and warrant further study. To further address the reviewer’s concern, we have added a statement to the Limitations section noting that, as our data rely on self-report, gender differences in perception or interpretation may influence reporting patterns and should be considered when interpreting subgroup comparisons.

**Modified text (Limitations, lines 486-488):** “Finally, as our outcomes and exposures rely on self-report, sex differences in perception or interpretation may influence reporting patterns.”

7. Lack of Qualitative Context
   Absence of narrative or qualitative data limits depth and interpretability of the results in such a nuanced area.

**Response:** We appreciate this important point and agree that qualitative data would provide deeper insight into how gender discrimination shapes professional identity formation in medical education. While our large-scale dataset was purely quantitative, we revised the manuscript to incorporate findings from existing qualitative research that contextualize gender discrimination’s impact—particularly among women students. We also now explicitly highlight the lack of qualitative work examining these experiences among men and discuss how this limits interpretation of our findings. Additionally, we have strengthened the Conclusion by recommending intersectional qualitative and mixed-methods research to address these gaps and deepen understanding of these complex dynamics (see revised Discussion, lines 400–412 and 426-433, and Conclusion, lines 524-527).

**Modified text:** Please see response to Reviewer 1’s comment #2

8. Generic Recommendations
   Conclusions lack specific, actionable strategies for institutions; practical implications are underdeveloped.

**Response:** We appreciate this helpful suggestion and have revised the “Implications” section, now titled “Implications and Recommendations”, to provide clearer, actionable strategies for institutions. Our recommendations include: (1) implementing implicit bias training for hiring committees, faculty, and students; (2) fostering belonging by transforming institutional culture to value and recognize contributions from marginalized learners; (3) using validated tools such as the PRODIGIE tool developed by our team to measure and improve the medical school learning environment; (4) increasing representation of women and gender-diverse individuals in academic medicine leadership; and (5) adopting formal anti-harassment policies that clearly define unacceptable behaviors. These revisions appear in the “Implications and Recommendations” section (lines 490-512).

**Modified text:** Please see response to Reviewer 1’s comment #3
