# Supplementary material for: Gender discrimination and personal and professional development fostered by allopathic medical schools in the United States
Source: PLoS One. 2026 Jun 22;21(6):e0319549. doi: 10.1371/journal.pone.0319549 (PMC13286186; doi:10.1371/journal.pone.0319549)
Supplement: Attachment — Submitted filename: Response to Reviewers_Nov 10.docx [file pone.0319549.s014.docx]

|  | **Department of Emergency Medicine** |
| --- | --- |
|  | Translational Research Building |
|  | 227 East 30th Street, 1st floor, NY, 10016 |
|  | T 475-241-5322  E [shruthi.venkataraman@nyulangone.org](mailto:shruthi.venkataraman@nyulangone.org) |
|  |  |

November 10, 2025

Sreeram V. Ramagopalan, PhD, MBA
Academic Editor
PLOS ONE

Dear Dr. Ramagopalan,

Thank you for you review of our manuscript, “Gender Discrimination and Medical Student Development.” We appreciate the reviewers’ thoughtful comments and recommendations and have responded to each concern and suggestion below:

**JOURNAL REQUIREMENTS:**

1. If the reviewer comments include a recommendation to cite specific previously published works, please review and evaluate these publications to determine whether they are relevant and should be cited. There is no requirement to cite these works unless the editor has indicated otherwise.

Response: We reviewed all reviewer comments carefully. None included recommendations to cite specific previously published works.

**REVIEWER’S COMMENTS**

**Reviewer #1:** 

1. I would like to thank the authors of the manuscript for making the requested corrections. It seems that the authors took a lot of time and care to make the corrections, which is appreciated. However, the following needs serious attention:

The authors stated regarding the validity and reliability of the instrument that "While not part of a validated scale, they exhibit strong face validity as direct, interpretable indicators of serious forms of gender discrimination in medical school settings."

If the instrument is not standardized, the manuscript and its data are not reliable. Were validity and reliability calculated for your study?

So I chose "Major Revision" to emphasize the importance of this issue.

Although these two tools were developed by a reputable and well-known scientific source, they need to be validated for your study population and context.

**Response:** We thank the reviewer for emphasizing the importance of measurement validity and reliability. We have now evaluated the internal consistency of the three AAMC Graduation Questionnaire (GQ) items assessing gender discrimination among respondents with complete data on all three items. Cronbach’s α = 0.58, indicating moderate inter-item coherence. This level of internal consistency is consistent with expectations for a brief, formative index designed to capture distinct manifestations of gender-based mistreatment (denied opportunities, sexist remarks, and biased evaluations). Because these items represent a formative rather than reflective construct, high inter-item correlations are not theoretically required.

Regarding validity, although no formal psychometric validation of these specific items has been published, they have been used consistently in multiple peer-reviewed national studies to assess gender discrimination and related outcomes among U.S. medical students (Nguyen et al., JAMA Pediatrics, 2022; Nguyen et al., JAMA Network Open, 2025). Their reproducible associations with key educational outcomes across diverse cohorts provide indirect evidence of construct validity. Moreover, the items are derived from a nationally administered instrument that undergoes annual expert review to ensure content relevance and clarity, supporting their face and content validity within this study context.

We have incorporated this clarification into the Methods section.

References:

Nguyen M, Chaudhry SI, Desai MM, Chen C, McDade WA, Fancher TL, Boatright D. Association of mistreatment and discrimination with medical school attrition. JAMA pediatrics. 2022 Sep 1;176(9):935-7.

Nguyen M, Venkataraman S, Abrams G, Pereira-Lima K, Fancher T, Addams AN, Moreland CJ, Boatright DH, Meeks LM. Discrimination Experiences Among Medical Students. JAMA Network Open. 2025 Oct 1;8(10):e2537871-.

**Modified text (lines 199-206):** “Internal consistency of the three gender discrimination items was assessed among respondents with complete data on all three items. Cronbach’s α = 0.58, indicating moderate inter-item coherence. This level of consistency is expected for a brief, three-item measure that captures distinct manifestations of gender-based mistreatment (denied opportunities, sexist remarks, and biased evaluations). Because these items represent a formative index rather than a reflective scale, high internal consistency is not theoretically required. The same AAMC GQ items have been used in multiple national studies examining discrimination among U.S. medical students. [11,12]”

**Reviewer #2:**

1. Sex vs. Gender Measurement:

The manuscript relies on a binary “sex” variable from AMCAS that may represent sex assigned at birth or gender identity. This ambiguity could introduce misclassification bias and should be more thoroughly emphasized.

**Response:** We agree and now emphasize the ambiguity of the AMCAS “Sex” item and its implications. We retain the term “sex” to match the instrument label but clarify in a clear and transparent manner that responses may reflect sex and/or gender identity in both the “Methods” and “Limitations” sections. We explicitly note potential nondifferential misclassification and its implication of biasing associations to the null.

**Modified text (lines 168-173): “**The item was labeled ‘Sex’ on the 2014 and 2015 AMCAS application and was not accompanied by a definition or clarification about whether it referred to sex assigned at birth or gender identity. As such, this variable may reflect sex, gender identity, or a combination thereof, depending on how applicants interpreted the question. We therefore interpret analyses as stratified by the AMCAS ‘Sex’ response and acknowledge potential nondifferential misclassification.”

**Modified text (lines 474-484):** “Although we refer to this variable as ‘sex’ to match the instrument label, the AMCAS item lacked a definition, which may have led some respondents to interpret it as gender identity, introducing potential misclassification that is likely nondifferential with respect to the outcomes and would bias associations toward the null. Prior work suggest that, in the absence of clarification, respondents may conflate sex and gender when completing survey items.[43] Similarly, the absence of sexual orientation data for our study years limits our ability to explore how intersecting sexual minority status (lesbian, gay, or bisexual identity), may influence personal and professional development. Together, these data limitations restrict the inclusivity and applicability of our findings and highlight the need for future research that captures a broader spectrum of sex, gender, and sexual orientation in medical education.”

2. Lack of Psychometric Validation:

The three AAMC Graduation Questionnaire items used to measure gender discrimination and the two items for personal/professional development have face validity but no established psychometric validation. This limits the reliability of the constructs.

**Response:** We added an internal-consistency check for the three GQ discrimination items (Cronbach’s α = 0.58 using respondents with complete data on all three items). We explain that this brief set functions as a formative index capturing distinct manifestations of gender discrimination; therefore, high α is not expected. For the PPIF items, we explicitly state that reliability coefficients are not applicable to single items, and that these items function as global indicators of perceived institutional support. We cite prior national studies employing the same discrimination and PPIF items.

**Modified text (lines 199-206):** “Internal consistency of the three gender discrimination items was assessed among respondents with complete data on all three items. Cronbach’s α = 0.58, indicating moderate inter-item coherence. This level of consistency is expected for a brief, three-item measure that captures distinct manifestations of gender-based mistreatment (denied opportunities, sexist remarks, and biased evaluations). Because these items represent a formative index rather than a reflective scale, high internal consistency is not theoretically required. The same AAMC GQ items have been used in multiple national studies examining discrimination among U.S. medical students.[11,12]”

**Modified text (lines 218-221):** “While they are not components of a validated scale and cannot be tested for reliability as single items, they are used in national research and institutional evaluation as global indicators of perceived institutional support for personal and professional development.[14,15]”

3. Cross-sectional Design:

The retrospective cross-sectional design precludes causal inference. The possibility of reverse causality (e.g., lower development perceptions influencing how students interpret discrimination) is not fully addressed.

**Response:** We thank the reviewer for this astute observation. We agree that we cannot infer causality and have now moreover clarified that temporal ordering cannot be established; perceptions of development could plausibly shape reporting of discrimination. We interpret estimates as associations consistent with theory and prior qualitative work and call for longitudinal mixed-methods.

**Modified text (lines 505-508):** “Finally, given the retrospective cross-sectional design, temporal ordering cannot be established, and reverse causality is possible (e.g., lower perceived development could influence interpretation/reporting of discrimination experiences). Associations should therefore be interpreted as non-causal.”

4. High Missing Data and Imputation:

Key variables (income, discrimination, outcomes) had large amounts of missing data (up to ~27%), and multiple imputation was used. While sensitivity analyses are reported, more discussion of how missingness may bias results would strengthen the paper.

**Response:** We thank the reviewer for raising this important point. We’ve now expanded the description of our multiple imputation strategy in the “Methods” section and added a statement on how missingness may bias results in the “Limitations” section. The imputation model included variables predictive of missingness and of the outcomes, and diagnostics compared observed versus imputed distributions. Complete-case results were directionally and numerically similar, reducing concern for bias from listwise deletion.

**Modified text (lines 231-236):** “The multiple imputation model included all predictors and outcomes as well as indicators plausibly related to missingness. Twenty imputed data sets were created. We assumed data were missing at random conditional on included variables. Diagnostics demonstrated model convergence and close alignment between observed and imputed marginal distributions (available on request). Complete-case analyses (n=21,728) yielded estimates similar in direction and magnitude (available on request).”

**Modified text (lines 490-492):** “Further, residual bias may persist if data were not missing at random. For instance, underrepresentation of students experiencing discrimination could lead to conservative estimates of its association with educational outcomes.”

5. Generalisability Outside the U.S.:

Findings are based on U.S. medical schools, and although the discussion briefly mentions other countries, external generalisability is limited.

**Response:** We agree and now state explicitly that external generalisability is limited in the discussion paragraph on “Applicability to other contexts”. This paragraph was originally added in response to prior reviewer feedback requesting consideration of this study’s relevance to other contexts and cultures in the “Discussion” section.

**Modified text (lines 457-460):** “Our study draws on data from U.S. medical schools and may have limited generalizability to medical educational settings outside North America where sociocultural norms, gender dynamics, and institutional structures differ and may shape both the experience and perceived impact of gender discrimination in distinct ways.

6. Limited Representation of Gender-diverse Students:

Non-binary, intersex, and gender-diverse students were not included due to data limitations. This restricts inclusivity and the applicability of findings.

**Response:** We agree and have further emphasized this limitation borne from AAMC data unavailability for our study years and proposed this line of enquiry as a future direction.

**Modified text (lines 472-484):** “Our binary sex variable (female/male) does not capture intersex variations. For our study years, the AAMC did not release data on intersex, non-binary, or other gender-diverse individuals because of the small sample sizes and concerns about maintaining anonymity. Although we refer to this variable as ‘sex’ to match the instrument label, the AMCAS item lacked a definition, which may have led some respondents to interpret it as gender identity, introducing potential misclassification that is likely nondifferential with respect to the outcomes and would bias associations toward the null. Prior work suggest that, in the absence of clarification, respondents may conflate sex and gender when completing survey items.[43] Similarly, the absence of sexual orientation data for our study years limits our ability to explore how intersecting sexual minority status (lesbian, gay, or bisexual identity), may influence personal and professional development. Together, these data limitations restrict the inclusivity and applicability of our findings and highlight the need for future research that captures a broader spectrum of sex, gender, and sexual orientation in medical education.”

7. Reliance on Self-report:

Both exposures (discrimination) and outcomes (development) are based on self-report, which may be influenced by recall bias, perception differences, or social desirability bias.

**Response:** We appreciate these important points and have now explicitly acknowledged the stated biases associated with self-reported data.

**Modified text (lines 503-505):** “Moreover, as our outcomes and exposures rely on self-report, recall bias, sex differences in perception or interpretation, and social desirability bias may influence reporting patterns.”

8. Narrow Outcome Measures:

Personal and professional identity formation (PPIF) is reduced to two single items, which may not capture the multidimensional and dynamic nature of identity formation.

**Response:** We agree and now explicitly acknowledge this limitation.

**Modified text (lines 497-500): “**Each outcome was derived from a single survey item that, while used nationally as a global indicator of institutional support, is not part of a validated multidimensional scale. Consequently, these measures may not encompass the complex, dynamic, and individualized nature of personal and professional identity formation.”

9. Limited Consideration of Intersectionality:

While race/ethnicity and income were adjusted for, the intersection of gender with race, sexual orientation, or socioeconomic status is not deeply explored. This may obscure important subgroup differences.

**Response:** We appreciate this thoughtful comment and agree that intersectional approaches are essential for understanding how multiple systems of marginalization jointly shape students’ experiences and development. Our primary aim in this study was to examine associations between gender discrimination and identity formation, and intersectional analyses, though important, were beyond the current scope. To acknowledge this point, we revised the opening of the “Implications” section to emphasize the need for future work that investigates how gender discrimination interacts with other axes of marginalization.

**Modified text (lines 510-514):** “Our findings highlight a persisting need for systemic reforms in medical education to address gender discrimination that is experienced by both female and male students, and to expand inquiry into how such discrimination intersects with broader axes of marginalization—rooted in racialization, class, and sexuality—to shape personal and professional identity formation and educational trajectories, including among gender-diverse students.”

10. Potential Cohort Effect:

The study spans students from 2014–2020. Cultural changes over time (e.g., #MeToo movement, institutional reforms) may have influenced responses, but temporal trends are not examined.

**Response:** We appreciate this comment and agree that cultural and institutional changes during the study period could have contributed to cohort differences. To account for potential temporal variation, we re-estimated all models including indicator terms for Graduation Questionnaire year. The results were substantively unchanged, suggesting that cohort-related differences did not meaningfully influence the observed associations.

**Modified text (lines 252-253):** “Sensitivity analyses including indicator terms for GQ year yielded results consistent with the main models, suggesting minimal influence of cohort effects.”

11. Overinterpretation of Male vs. Female Differences:

The paper emphasizes that males showed sharper declines in PPIF with discrimination, but this interpretation may be speculative without qualitative evidence to support mechanisms.

**Response:** We appreciate this comment and agree that causal mechanisms cannot be inferred from quantitative data alone. We have revised the text to clarify that our interpretation of the observed sex differences is cautious and informed by prior empirical and theoretical work. We also emphasize the limited qualitative research on men’s experiences of gender discrimination in medical education, which constrains interpretation and highlights the need for future intersectional qualitative inquiry.

**Modified text (lines 421-423):** “While the mechanisms underlying this pattern cannot be determined from our data, we interpret these findings cautiously in light of prior empirical and theoretical work.”

12. Heavy Tables/Figures:

Some tables and figures are very dense and could be simplified or moved to supplementary material for clarity.

**Response:** We appreciate this suggestion. To improve clarity and reduce visual density, we now present only the imputed-dataset descriptives in Table 1, while the complete-case descriptives have been moved to the Supplement (eTable S1). All four figures were retained because each serves a distinct analytic function essential to interpreting the study’s central question. Figure 1 depicts the distribution of gender-discrimination exposure by sex, providing necessary context for subsequent models. Figure 2 shows overall sex differences in personal and professional development, establishing the baseline association. Figure 3 presents sex-stratified dose–response relationships between discrimination frequency and each outcome, illustrating the graded pattern within males and females. Figure 4 quantifies the interaction by displaying predicted adjusted relative risks and percentage change (Δ) across discrimination levels, summarizing the magnitude of decline for each sex and clarifying how those slopes differ. Whereas Figure 3 visualizes the pattern of decline, Figure 4 isolates and quantifies the interaction effect, the comparative steepness of decline between sexes, derived from the joint model. Together, these figures form a sequential visual narrative that moves from exposure distribution to baseline differences, stratified associations, and finally, quantified interaction. Removing or relocating any of them would disrupt this analytic logic and obscure interpretation of the central effect modification.

13. Policy Recommendations Could Be Sharper:

While the manuscript calls for reforms, the recommendations remain somewhat general (e.g., “implicit bias training”). More concrete, evidence-based interventions would strengthen the implications.

**Response:** We appreciate this helpful feedback and have thoroughly revised the “Implications and Recommendations” section to strengthen the specificity and evidence base of our proposed reforms. The revised text now grounds each recommendation in empirical or review literature, expanding beyond general references to implicit bias training to include multilevel, actionable strategies.

**Modified text (lines 510-555):** “Our findings highlight a persisting need for systemic reforms in medical education to address gender discrimination that is experienced by both female and male students, and to expand inquiry into how such discrimination intersects with broader axes of marginalization—rooted in racialization, class, and sexuality—to shape PPIF and educational trajectories, including among gender-diverse students. Addressing gender discrimination in the learning environment, as well as racial discrimination, as our previous work suggests, may serve to promote holistic development for all students.[44] We propose considering the integration of PPIF as a critical equity metric and formally including PPIF parity for students of all racial, ethnic, and gender backgrounds in the LCME accreditation standards.[45] In line with global recommendations for gender-transformative health professional education, medical schools should periodically conduct institutional “gender audits” to identify how entrenched gender norms, power hierarchies, and policies—collectively termed gender regimes—shape student experiences and perpetuate inequities.[46] Embedding such audits within accreditation and quality-improvement processes could ensure accountability for sustained gender equity.

Beyond brief stand-alone bias sessions, institutions should implement a coordinated set of actions that operate at individual, organizational, and structural levels. A successful example is Stanford University’s multifaceted gender climate initiative, which required all faculty to attend sexual-harassment prevention training; convened small-group leadership retreats to address gender and racial dynamics in supervision and evaluation; provided student-focused workshops on professional boundaries and reporting pathways; created a standing diversity council reporting directly to the Dean; and formalized clear, accessible procedures for addressing harassment complaints.[47] Building on this model, a systematic review recommended implementation of a “basic bundle” of interventions including (1) explicit institutional policies that clearly define prohibited behaviors and outline sanctions, (2) confidential and well-supported reporting mechanisms with guaranteed follow-up and protection from retaliation, and (3) structured educational programming tailored to different roles—including faculty evaluators, clinical supervisors, and students—to reinforce accountability and behavioral expectations.[48]These reforms should be complemented by measures addressing caregiver discrimination—such as flexible training schedules, protected parental leave, and accessible child-care supports—which have been identified as critical for retaining trainees and sustaining equitable professional identity development.[46]

Fostering a sense of belonging for minoritized and marginalized learners in medicine—by uncovering unconscious biases and transforming academic medicine environments to recognize, hear, and value their contributions—can positively influence PPIF in medical school.[49,50] Validated tools that can systematically measure and help achieve a climate of equity and inclusion within the medical school learning environment, such as the PRODIGIE tool developed by our team, may provide medical schools with actionable insights to foster equity and inclusion,[51] ultimately improving educational outcomes, including PPIF, for all students. Parallel structural supports—such as dedicated gender-equity or equal-opportunity offices that coordinate harassment prevention, caregiving policies, and climate data reporting—have been identified as foundational components of sustainable gender-transformative reform.[46] Complimentary structural interventions include increasing the representation of women and gender-diverse individuals in academic medicine leadership, and embedding climate and inclusion metrics in faculty promotion criteria [48,52,53]”

Thank you for considering our revised manuscript. We appreciate the reviewers’ thoughtful feedback and would be happy to provide any further clarification if needed. We look forward to your decision.

Respectfully,

Shruthi Venkataraman, MD, MS

Postdoctoral Research Fellow, Boatright Lab

NYU Grossman School of Medicine
