# Supplementary material for: Gender discrimination and personal and professional development fostered by allopathic medical schools in the United States
Source: PLoS One. 2026 Jun 22;21(6):e0319549. doi: 10.1371/journal.pone.0319549 (PMC13286186; doi:10.1371/journal.pone.0319549)
Supplement: Attachment — Submitted filename: Response_to_Reviewers_auresp_3.docx [file pone.0319549.s015.docx]

|  | **Department of Emergency Medicine** |
| --- | --- |
|  | Translational Research Building |
|  | 227 East 30th Street, 1st floor, NY, 10016 |
|  | T 475-241-5322  E [shruthi.venkataraman@nyulangone.org](mailto:shruthi.venkataraman@nyulangone.org) |
|  |  |

March 26, 2026

Sreeram V. Ramagopalan, PhD, MBA
Academic Editor
PLOS ONE

Dear Dr. Ramagopalan,

Thank you for you review of our manuscript, “Gender Discrimination and Medical Student Development.” We appreciate the reviewers’ thoughtful comments and recommendations and have responded to each concern and suggestion below:

**REVIEWER’S COMMENTS**

**Reviewer #1:**

1. The data provided about the questionnaire does not confirm its reliability and validity. In fact, gender discrimination was examined with three questions that have no quantitative and qualitative support (validity confirmation). The authors considered its validity and reliability to be confirmed because the questionnaire was developed by AAMC. These issues make the value of the data problematic. Cronbach's alpha indicates the reliability of the data, and factor analysis is needed to examine the validity. How were these three questions extracted?

**Response:** The three gender-discrimination items used in our study are part of the AAMC’s Graduation Questionnaire (GQ), and these exact items were incorporated into the validated PRODIGIE tool (Boatright et al., 2024). As shown in Boatright et al. (2024), these GQ discrimination items underwent exploratory factor analysis, confirmatory factor analysis, and internal-consistency testing within a national dataset of over 60,000 medical students. In the PRODIGIE validation, the GQ discrimination factor (“Discrimination: race, ethnicity, and gender”) demonstrated acceptable psychometric performance with factor loadings ≥0.43 and Cronbach’s α = 0.76 (Boatright et al., 2024). Thus, the validity and reliability of these items have been established in prior psychometric evaluation. In our manuscript, we additionally reported Cronbach’s α for these three items gender discrimination items in our dataset, as previously requested.

**Reference:** Boatright, D., Nguyen, M., Hill, K., Berg, D., Castillo-Page, L., Anderson, N., ... & White, M. A. (2024). Development of a tool to measure student perceptions of equity and inclusion in medical schools. JAMA Network Open, 7(2), e240001.

***Modified text (lines 195-203):*** *“These items have been psychometrically validated as part of the PRODIGIE tool using the same 2016–2020 GQ cohorts analyzed in our study, undergoing exploratory and confirmatory factor analyses and demonstrating acceptable internal consistency for the discrimination factor (Cronbach α = 0.76).[11] While these items were validated within the broader discrimination factor in PRODIGIE, we use them here as a formative index capturing distinct manifestations of gender‑based mistreatment, for which high internal consistency is not required. When assessed in our sample, the three items demonstrated a Cronbach’s α of 0.58, which is expected for a brief formative index.”*

2. Also, two questions (one question related to personal development and one question related to professional development) were used to assess personal and professional development. While there are standard tools for assessing professional identity, two questions alone are not a complete criterion for assessing professional identity. In other words, the relationship between different questions in a questionnaire was used to examine the two variables of gender discrimination and professional identity.

**Response:** We agree that the two GQ items used to assess personal and professional development are not part of a validated multi‑item scale and that they are not a complete criterion for assessing professional identity formation and have acknowledged this as a limitation (see lines 508-517). These items are single‑item indicators that are used nationally by the AAMC for institutional assessment and have been used in prior peer‑reviewed research for this purpose (Venkataraman et al., 2024, Venkataraman et al., 2026). Because they are single items, psychometric analyses such as internal consistency or factor analysis are not applicable. In the manuscript, we analyze these items separately and do not treat them as a scale, consistent with prior studies using these same GQ items. We have now amended the text, including the title of the manuscript, to make it clear that personal and professional development are two separate constructs that were measured separately.

**References:**

1) Venkataraman, S., Nguyen, M., Chaudhry, S. I., Desai, M. M., Hajduk, A. M., Mason, H. R., ... & Boatright, D. (2024). Racial and ethnic discrimination and medical students’ identity formation. JAMA Network Open, 7(10), e2439727.
2) Venkataraman, S., Nguyen, M., Hajduk, A. M., Ayedun, A., Roberts, W., Shanab, B., ... & Boatright, D. (2026). Meaningful Interactional Diversity, Professional Development, and Service Intent in White Medical Students. JAMA Network Open, 9(2), e2560266.

***Modified text (line 1):*** *“Gender discrimination and personal and professional development fostered by medical schools”*

***Modified text (lines 48-50):*** *“PPIF was assessed using two separate GQ metrics assessing student agreement on a 5-point Likert scale that their medical school fostered and nurtured their development as a person and a future physician, respectively, and dichotomized.”*

***Associated text (line 206):*** *“PPIF was assessed using two distinct metrics: personal and professional development.”*

***Associated text (lines 213-215):*** *“Both personal and professional development were examined separately in all analyses; the term PPIF is used to collectively refer to these two outcomes.”*

3. The study analyzed data between 2015 and 2020, while we are now in 2026, and many factors have affected the two variables above. Given the changing academic generations, the above conclusion may be affected.

**Response:** The study period (2016–2020) reflects the years for which all required GQ variables were available in analyzable form when this study was conducted. Because the reviewer’s concern relates to potential changes across academic years, we evaluated this directly by conducting sensitivity analyses that included indicator terms for GQ year. These analyses produced estimates that were highly consistent with the main models, indicating that associations did not vary meaningfully across cohorts in the available data. Nonetheless, we acknowledge that patterns should be reexamined in more recent cohorts, and have noted this as a future direction.

***Modified text (lines 579-583):*** *“Future research is needed to understand how gender discrimination influences the holistic development of medical students across the spectrum of gender identity and expression—including in more recent national cohorts—and through longitudinal qualitative and mixed methods approaches that include women, men, and gender-diverse students with intersecting marginalized identities.”*

4. It would have been reliable and valuable if the researchers had first examined the psychometrics of the questionnaire, identified the factors, and then examined this issue in graduates of different years

**Response:** The discrimination items used in our study have already undergone psychometric evaluation in prior work (please see response to Comment #1). As part of the PRODIGIE validation, these same GQ discrimination items were examined using the 2016–2020 AAMC Graduation Questionnaire cohorts—the identical GQ years included in our study—and underwent exploratory and confirmatory factor analyses as well as internal‑consistency testing (Boatright et al., 2024). Because these items have already been validated using the same national GQ administrations, additional psychometric analyses were not required for the present study.

Regarding the reviewer’s point about “graduates of different years,” we evaluated potential cohort differences directly by conducting sensitivity analyses that included indicator terms for GQ year. These analyses produced estimates consistent with the main models, indicating no evidence of cohort‑related variation in the associations for the years examined.

**Reviewer #3:**

1. Line 225: The total percentage missing data to be stated.

**Response:** We have now added this information to the ‘Methods’ section.

***Modified text (lines 226-227):*** *“A total of 21,728 participants (57.77%) answered all the questionnaire items relevant to this study.”*

2. Line 229: For the sentence ‘Missing data were imputed using a fully conditional specification method to handle arbitrary missing patterns across all categorical data.', what about the continuous data and what sort of statistical approach was used?

**Response:** There were no continuous variables included in this analysis.

3. Line 252-253: The statement ‘Sensitivity analyses including indicator terms for GQ year yielded results consistent with the main models, suggesting minimal influence of cohort effects.’ is to be placed in the results section. If were to place in the methods section, it should state ‘Sensitivity analyses including indicator terms for GQ year were conducted to assess potential cohort effects.’

**Response:** The phrasing has been changed as advised in the ‘Methods’ section, and a statement of the results of the sensitivity analyses was included in the ‘Results’ section.

***Modified text (lines 250-251):*** *“Sensitivity analyses including indicator terms for GQ year were conducted to assess potential cohort effects.”*

***Modified text (lines 356-357):*** *“Across analyses, adding GQ-year indicator terms in sensitivity analyses produced results consistent with the main models.”*

4. Line 233: MAR is to be placed in bracket for missing at random.

Response: This addition was made.

***Modified text (lines 230-231):*** *“We assumed data were missing at random (MAR) conditional on included variables.”*

5. All statistical tests used in this study are to be reported in the Statistical Analyses section.

**Response:** We have ensured that all statistical tests used in this study are reported in the “Statistical Analysis” subsection of the “Methods” section.

6. Table 1: 'Other' is to be placed after ‘White’ category.

**Response:**; The proposed change has now been made, however, please note that the groups are no longer listed in alphabetical order.

***Modified text (line 258)):*** *Please see Table 1.*

7. Statistical results and figures in the Results section are to be presented in tables to provide detailed information and to facilitate comparison and interpretation. Some tables may be included as supplementary files.

**Response:** Thank you for this helpful suggestion. In response, we have created a complete set of detailed tables corresponding to each results figure. These tables provide all underlying numerical values, including Ns, percentages, and adjusted relative risks with 95% confidence intervals. As recommended, we have included these tables as Supporting Information (S1–S8 Tables), with each table clearly linked to the figure it corresponds to (e.g., “corresponds to Figure 3”).

8. The detailed table output including interaction term derived from GLM is to be presented.

**Response:** We have added the full generalized linear model output for both outcomes. The S9 Table (personal development) and S10 Table (professional development) report all coefficients from the Poisson GLM, including the sex × gender‑discrimination interaction terms, main‑effect terms, and all covariates. Each table includes the IRR, robust standard error, z‑statistic, p‑value, and 95% confidence interval, providing the exact interaction parameters estimated by the model.

Thank you for considering our revised manuscript. We appreciate the reviewers’ thoughtful feedback and would be happy to provide any further clarification if needed. We look forward to your decision.

Respectfully,

Shruthi Venkataraman, MD, MS

Postdoctoral Research Fellow, Boatright Lab

NYU Grossman School of Medicine
