# Supplementary material for: Gender discrimination and personal and professional development fostered by allopathic medical schools in the United States
Source: PLoS One. 2026 Jun 22;21(6):e0319549. doi: 10.1371/journal.pone.0319549 (PMC13286186; doi:10.1371/journal.pone.0319549)
Supplement: Attachment — Submitted filename: Response_to_Reviewers_auresp_4.docx [file pone.0319549.s016.docx]

|  | **Department of Emergency Medicine** |
| --- | --- |
|  | Translational Research Building |
|  | 227 East 30th Street, 1st floor, NY, 10016 |
|  | T 475-241-5322  E [shruthi.venkataraman@nyulangone.org](mailto:shruthi.venkataraman@nyulangone.org) |
|  |  |

May 24, 2026

Sreeram V. Ramagopalan, PhD, MBA
Academic Editor
PLOS ONE

Dear Dr. Ramagopalan,

Thank you for you review of our manuscript, “Gender Discrimination and Medical Student Development.” We have addressed Reviewer 3’s comments below:

**REVIEWER’S COMMENTS**

**Reviewer #3:**

1. Supplementary tables: Technically, the p-value cannot be zero; use 'p < ..' instead.

**Response**: Thank you for this important flag. We’ve changed the p values in supplementary tables 7 to 10 to non-zero values. If the p value is less than 0.001, we’ve indicated it as such, rather report it incorrectly as zero.

***Modified text****: Please see p-value reporting in supplementary tables 7-10 (S7 Table to S10 Table).*

2. Supplementary tables: Exp(B) is to be replaced with 'OR'.

Response: Given that our models used a Poisson distribution with robust standard errors (a method specifically chosen for the common, binary outcomes in our study, a context in which odd’s ratios [ORs] are known to overestimate the magnitude of association compared to relative risks; *see Zou, 2004*), the exponentiated coefficients from these regression models represent adjusted relative risks (aRRs) rather than ORs. As such, we have replaced the “exp(b)” with “aRR”. We have also cited the ‘Zou, 2004’ reference to the ‘Statistical Analysis’ section of the ‘Methods’.

***Modified text****: We have replaced “exp(b)” with “aRR” in supplementary tables 7 and 8 (S7 Table and S8 Table). We have also updated the effect measure column header from "IRR" to "aRR" in supplementary tables 9 and 10 (S9 Table and S10 Table) for consistency.*

***Modified text (lines 60, 347, 353, 357)****: We have replaced instances of “exp(b)” in the manuscript text with “aRR”.*

***Modified text (lines 239-240)****: “We used this method because PPIF outcomes were common. [19,20]”*

***Reference****: Zou, G. (2004). A modified Poisson regression approach to prospective studies with binary data. American journal of epidemiology, 159(7), 702-706.*

3. Supplementary tables: Use the format '95% CI (lower–upper)' instead of separate columns for '95% CI Lower Limit' and '95% CI Upper Limit'.

**Response**: We have now used the suggested format to report 95% CIs in supplementary tables 3 to 10.

***Modified text:*** *Please see the 95% CI reporting in supplementary tables 3 to 10 (S3 Table to S10 Table).*

4. Supplementary tables: The tables columns width is to be adjusted.

**Response**: We have now adjusted the column widths of all the supplementary tables to ensure readability.

***Modified text:*** *Please see supplementary tables 1 to 10 (S1 Table to S10 Table).*

Thank you for considering our revised manuscript. We greatly appreciate the thought and consideration you and the reviewers have given to strengthening this manuscript and remain available to provide any further clarification. We look forward to your decision.

Sincerely,

Shruthi Venkataraman, MD, MS

Postdoctoral Research Fellow, Boatright Lab

NYU Grossman School of Medicine
