# Supplementary material for: Gender discrimination and personal and professional development fostered by allopathic medical schools in the United States
Source: PLoS One. 2026 Jun 22;21(6):e0319549. doi: 10.1371/journal.pone.0319549 (PMC13286186; doi:10.1371/journal.pone.0319549)
Supplement: Peer review history

# Peer review: Decision Letter 0

**DOI:** 10.1371/journal.pone.0319549.r001

**Authors:** Somayeh Delavari

27 May 2025

PONE-D-25-05663

Gender Discrimination and Medical Student Development

PLOS ONE

Dear Dr. Venkataraman,

Thank you for submitting your manuscript to PLOS ONE. After careful consideration, we feel that it has merit but does not fully meet PLOS ONE’s publication criteria as it currently stands. Therefore, we invite you to submit a revised version of the manuscript that addresses the points raised during the review process.

Please submit your revised manuscript by Jul 10 2025 11:59PM. If you will need more time than this to complete your revisions, please reply to this message or contact the journal office at plosone@plos.org. When you're ready to submit your revision, log on to https://www.editorialmanager.com/pone/ and select the 'Submissions Needing Revision' folder to locate your manuscript file.

Please include the following items when submitting your revised manuscript:

- A rebuttal letter that responds to each point raised by the academic editor and reviewer(s). You should upload this letter as a separate file labeled 'Response to Reviewers'.
- A marked-up copy of your manuscript that highlights changes made to the original version. You should upload this as a separate file labeled 'Revised Manuscript with Track Changes'.
- An unmarked version of your revised paper without tracked changes. You should upload this as a separate file labeled 'Manuscript'.

If you would like to make changes to your financial disclosure, please include your updated statement in your cover letter. Guidelines for resubmitting your figure files are available below the reviewer comments at the end of this letter.

If applicable, we recommend that you deposit your laboratory protocols in protocols.io to enhance the reproducibility of your results. Protocols.io assigns your protocol its own identifier (DOI) so that it can be cited independently in the future. For instructions see: https://journals.plos.org/plosone/s/submission-guidelines#loc-laboratory-protocols. Additionally, PLOS ONE offers an option for publishing peer-reviewed Lab Protocol articles, which describe protocols hosted on protocols.io. Read more information on sharing protocols at https://plos.org/protocols?utm_medium=editorial-email&utm_source=authorletters&utm_campaign=protocols.

We look forward to receiving your revised manuscript.

Kind regards,

Somayeh Delavari, Ph.D.,

Academic Editor

PLOS ONE

**Journal Requirements:**

1. When submitting your revision, we need you to address these additional requirements.

Please ensure that your manuscript meets PLOS ONE's style requirements, including those for file naming. The PLOS ONE style templates can be found at

https://journals.plos.org/plosone/s/file?id=wjVg/PLOSOne_formatting_sample_main_body.pdf and

https://journals.plos.org/plosone/s/file?id=ba62/PLOSOne_formatting_sample_title_authors_affiliations.pdf

2. Thank you for stating the following financial disclosure:

Alexis Webber, Mytien Nguyen, Hyacinth RC Mason, Mayur M. Desai, and Shruthi Venkataraman report no conflicts of interest. Dowin Boatright received NIH grant (R35GM153263) support. Alexandra Hajduk received NIH grants (R01 GM146147, R01 MD018928, R01 HL160822) and Burroughs-Welcome Fund travel support. Mytien received NIGMS (T32GM136651) and NIAID (F30AI157227) grant support. Sarwat Chaudhry received support from NIH/DHHS (1R01MD018928-01A1) and NIH/NIGMS (GM146147). Tonya L. Fancher received grants and consulting fees from the American Medical Association, honoraria from Montefiore School of Medicine, Yale School of Medicine, University of Minnesota School of Medicine, travel support from ACGME, and holds a leadership role with HRSA.

Please state what role the funders took in the study.  If the funders had no role, please state: "The funders had no role in study design, data collection and analysis, decision to publish, or preparation of the manuscript."

If this statement is not correct you must amend it as needed.

Please include this amended Role of Funder statement in your cover letter; we will change the online submission form on your behalf.

3. Thank you for stating the following in the Acknowledgments Section of your manuscript:

This work is supported by the National Institute of Health: NIGMS grant T32GM136651 (MN), NIAID grant F30AI157227 (MN), NIGMS grant R35GM153263 (DB). The funder had no role in the design and conduct of the study; collection, management, analysis, and interpretation of the data; preparation, review, or approval of the manuscript; and decision to submit the manuscript for publication. This material is based upon data provided by the Association of American Medical Colleges (“AAMC”). The views expressed herein are those of the authors and do not necessarily reflect the position or policy of the AAMC.

We note that you have provided funding information that is not currently declared in your Funding Statement. However, funding information should not appear in the Acknowledgments section or other areas of your manuscript. We will only publish funding information present in the Funding Statement section of the online submission form.

Please remove any funding-related text from the manuscript and let us know how you would like to update your Funding Statement. Currently, your Funding Statement reads as follows:

Alexis Webber, Mytien Nguyen, Hyacinth RC Mason, Mayur M. Desai, and Shruthi Venkataraman report no conflicts of interest. Dowin Boatright received NIH grant (R35GM153263) support. Alexandra Hajduk received NIH grants (R01 GM146147, R01 MD018928, R01 HL160822) and Burroughs-Welcome Fund travel support. Mytien received NIGMS (T32GM136651) and NIAID (F30AI157227) grant support. Sarwat Chaudhry received support from NIH/DHHS (1R01MD018928-01A1) and NIH/NIGMS (GM146147). Tonya L. Fancher received grants and consulting fees from the American Medical Association, honoraria from Montefiore School of Medicine, Yale School of Medicine, University of Minnesota School of Medicine, travel support from ACGME, and holds a leadership role with HRSA.

Please include your amended statements within your cover letter; we will change the online submission form on your behalf.

4. In the online submission form, you indicated that your data is available only on request from a third party. Please note that your Data Availability Statement is currently missing the contact details for the third party, such as an email address or a link to where data requests can be made. Please update your statement with the missing information.

[Note: HTML markup is below. Please do not edit.]

Reviewers' comments:

Reviewer's Responses to Questions

**Comments to the Author**

1. Is the manuscript technically sound, and do the data support the conclusions?

The manuscript must describe a technically sound piece of scientific research with data that supports the conclusions. Experiments must have been conducted rigorously, with appropriate controls, replication, and sample sizes. The conclusions must be drawn appropriately based on the data presented.

Reviewer #1: Partly

Reviewer #2: Yes

**********

2. Has the statistical analysis been performed appropriately and rigorously?

Reviewer #1: Yes

Reviewer #2: Yes

**********

3. Have the authors made all data underlying the findings in their manuscript fully available?

The PLOS Data policy requires authors to make all data underlying the findings described in their manuscript fully available without restriction, with rare exception (please refer to the Data Availability Statement in the manuscript PDF file). The data should be provided as part of the manuscript or its supporting information, or deposited to a public repository. For example, in addition to summary statistics, the data points behind means, medians and variance measures should be available. If there are restrictions on publicly sharing data—e.g. participant privacy or use of data from a third party—those must be specified.

Reviewer #1: No

Reviewer #2: Yes

**********

4. Is the manuscript presented in an intelligible fashion and written in standard English?

PLOS ONE does not copyedit accepted manuscripts, so the language in submitted articles must be clear, correct, and unambiguous. Any typographical or grammatical errors should be corrected at revision, so please note any specific errors here.

Reviewer #1: Yes

Reviewer #2: Yes

**********

5. Review Comments to the Author

Please use the space provided to explain your answers to the questions above. You may also include additional comments for the author, including concerns about dual publication, research ethics, or publication ethics. (Please upload your review as an attachment if it exceeds 20,000 characters)

**Reviewer #1:**   Dear

The current study is a valuable study on gender discrimination. However, the following questions need further investigation:

Is this study applicable to other societies and cultures? This needs to be addressed in the discussion section.

The authors used existing data collected over time. It seems that a supplementary study in the form of interviews (qualitative study) is needed to support the data.

What do the authors suggest for improving the situation? What has been stated in similar studies?

The statement of the problem is not well explained. This needs to be addressed different articles and their differing views on this matter. There is a need to further explain the context of the study and also to explain the differences between the contexts on this issue.

The study tools and how to ensure its validity and reliability should be stated.

**Reviewer #2:**  1. Binary Gender Classification

The study excludes non-binary individuals, limiting the scope of analysis despite addressing "gender discrimination."

2. Weak Operationalization of PPIF

Personal and professional identity formation is measured via unvalidated single-item indicators, insufficient for such a complex construct.

3. Unclear Discrimination Categories

The classification of gender discrimination lacks methodological transparency and may not reflect actual experiential differences.

4. Insufficient Interaction Modeling

Analyses of gender × discrimination interaction effects are inadequately described and potentially underadjusted for confounders.

5. High Missing Data and Imputation Dependence

Heavy reliance on imputed data (up to 27% missingness) without detailed diagnostics weakens the credibility of findings.

6. Interpretation Risk: Male Vulnerability

Claims that men experience sharper declines in PPIF may reflect perception/reporting biases rather than true differential impact.

7. Lack of Qualitative Context

Absence of narrative or qualitative data limits depth and interpretability of the results in such a nuanced area.

8. Generic Recommendations

Conclusions lack specific, actionable strategies for institutions; practical implications are underdeveloped.

**********

6. PLOS authors have the option to publish the peer review history of their article (what does this mean?). If published, this will include your full peer review and any attached files.

If you choose “no”, your identity will remain anonymous but your review may still be made public.

**Do you want your identity to be public for this peer review?**   For information about this choice, including consent withdrawal, please see our Privacy Policy.

Reviewer #1: No

Reviewer #2: No

**********

[NOTE: If reviewer comments were submitted as an attachment file, they will be attached to this email and accessible via the submission site. Please log into your account, locate the manuscript record, and check for the action link "View Attachments". If this link does not appear, there are no attachment files.]

While revising your submission, please upload your figure files to the Preflight Analysis and Conversion Engine (PACE) digital diagnostic tool, https://pacev2.apexcovantage.com/. PACE helps ensure that figures meet PLOS requirements. To use PACE, you must first register as a user. Registration is free. Then, login and navigate to the UPLOAD tab, where you will find detailed instructions on how to use the tool. If you encounter any issues or have any questions when using PACE, please email PLOS at figures@plos.org. Please note that Supporting Information files do not need this step.

Attachment Submitted filename: PONE-D-25-05663.pdf

Attachment Submitted filename: Binary Gender Classification.docx

# Author comment: Author response to Decision Letter 1

**DOI:** 10.1371/journal.pone.0319549.r002

10 Aug 2025

Thank you for the opportunity to revise our manuscript. We have uploaded the revised manuscript, a marked-up copy with tracked changes, our detailed response to reviewers, and figures verified via the PACE tool to ensure compliance with PLOS ONE requirements.

Attachment Submitted filename: Response to Reviewers.docx

# Peer review: Decision Letter 1

**DOI:** 10.1371/journal.pone.0319549.r003

**Authors:** Somayeh Delavari, Sreeram Ramagopalan

24 Sep 2025

PONE-D-25-05663R1

Gender Discrimination and Medical Student Development

PLOS ONE

Dear Dr. Venkataraman,

Thank you for submitting your manuscript to PLOS ONE. After careful consideration, we feel that it has merit but does not fully meet PLOS ONE’s publication criteria as it currently stands. Therefore, we invite you to submit a revised version of the manuscript that addresses the points raised during the review process.

Please submit your revised manuscript by Nov 08 2025 11:59PM. If you will need more time than this to complete your revisions, please reply to this message or contact the journal office at plosone@plos.org. When you're ready to submit your revision, log on to https://www.editorialmanager.com/pone/ and select the 'Submissions Needing Revision' folder to locate your manuscript file.

Please include the following items when submitting your revised manuscript:

- A rebuttal letter that responds to each point raised by the academic editor and reviewer(s). You should upload this letter as a separate file labeled 'Response to Reviewers'.
- A marked-up copy of your manuscript that highlights changes made to the original version. You should upload this as a separate file labeled 'Revised Manuscript with Track Changes'.
- An unmarked version of your revised paper without tracked changes. You should upload this as a separate file labeled 'Manuscript'.

If you would like to make changes to your financial disclosure, please include your updated statement in your cover letter. Guidelines for resubmitting your figure files are available below the reviewer comments at the end of this letter.

If applicable, we recommend that you deposit your laboratory protocols in protocols.io to enhance the reproducibility of your results. Protocols.io assigns your protocol its own identifier (DOI) so that it can be cited independently in the future. For instructions see: https://journals.plos.org/plosone/s/submission-guidelines#loc-laboratory-protocols. Additionally, PLOS ONE offers an option for publishing peer-reviewed Lab Protocol articles, which describe protocols hosted on protocols.io. Read more information on sharing protocols at https://plos.org/protocols?utm_medium=editorial-email&utm_source=authorletters&utm_campaign=protocols.

We look forward to receiving your revised manuscript.

Kind regards,

Sreeram V. Ramagopalan

Academic Editor

PLOS ONE

Journal Requirements:

If the reviewer comments include a recommendation to cite specific previously published works, please review and evaluate these publications to determine whether they are relevant and should be cited. There is no requirement to cite these works unless the editor has indicated otherwise.

[Note: HTML markup is below. Please do not edit.]

Reviewers' comments:

Reviewer's Responses to Questions

**Comments to the Author**

1. If the authors have adequately addressed your comments raised in a previous round of review and you feel that this manuscript is now acceptable for publication, you may indicate that here to bypass the “Comments to the Author” section, enter your conflict of interest statement in the “Confidential to Editor” section, and submit your "Accept" recommendation.

Reviewer #1: All comments have been addressed

Reviewer #2: All comments have been addressed

**********

2. Is the manuscript technically sound, and do the data support the conclusions?

The manuscript must describe a technically sound piece of scientific research with data that supports the conclusions. Experiments must have been conducted rigorously, with appropriate controls, replication, and sample sizes. The conclusions must be drawn appropriately based on the data presented.

Reviewer #1: Partly

Reviewer #2: Yes

**********

3. Has the statistical analysis been performed appropriately and rigorously?

Reviewer #1: Yes

Reviewer #2: Yes

**********

4. Have the authors made all data underlying the findings in their manuscript fully available?

The PLOS Data policy requires authors to make all data underlying the findings described in their manuscript fully available without restriction, with rare exception (please refer to the Data Availability Statement in the manuscript PDF file). The data should be provided as part of the manuscript or its supporting information, or deposited to a public repository. For example, in addition to summary statistics, the data points behind means, medians and variance measures should be available. If there are restrictions on publicly sharing data—e.g. participant privacy or use of data from a third party—those must be specified.

Reviewer #1: Yes

Reviewer #2: Yes

**********

5. Is the manuscript presented in an intelligible fashion and written in standard English?

PLOS ONE does not copyedit accepted manuscripts, so the language in submitted articles must be clear, correct, and unambiguous. Any typographical or grammatical errors should be corrected at revision, so please note any specific errors here.

Reviewer #1: Yes

Reviewer #2: Yes

**********

6. Review Comments to the Author

Please use the space provided to explain your answers to the questions above. You may also include additional comments for the author, including concerns about dual publication, research ethics, or publication ethics. (Please upload your review as an attachment if it exceeds 20,000 characters)

Reviewer #1: I would like to thank the authors of the manuscript for making the requested corrections. It seems that the authors took a lot of time and care to make the corrections, which is appreciated. However, the following needs serious attention:

The authors stated regarding the validity and reliability of the instrument that "While not part of a validated scale, they exhibit strong face validity as direct, interpretable indicators of serious forms of gender discrimination in medical school settings."

If the instrument is not standardized, the manuscript and its data are not reliable. Were validity and reliability calculated for your study?

So I chose "Major Revision" to emphasize the importance of this issue.

Although these two tools were developed by a reputable and well-known scientific source, they need to be validated for your study population and context.

Reviewer #2: Potential Weaknesses / Limitations

1. Sex vs. Gender Measurement:

The manuscript relies on a binary “sex” variable from AMCAS that may represent sex assigned at birth or gender identity. This ambiguity could introduce misclassification bias and should be more thoroughly emphasized.

2. Lack of Psychometric Validation:

The three AAMC Graduation Questionnaire items used to measure gender discrimination and the two items for personal/professional development have face validity but no established psychometric validation. This limits the reliability of the constructs.

3. Cross-sectional Design:

The retrospective cross-sectional design precludes causal inference. The possibility of reverse causality (e.g., lower development perceptions influencing how students interpret discrimination) is not fully addressed.

4. High Missing Data and Imputation:

Key variables (income, discrimination, outcomes) had large amounts of missing data (up to ~27%), and multiple imputation was used. While sensitivity analyses are reported, more discussion of how missingness may bias results would strengthen the paper.

5. Generalisability Outside the U.S.:

Findings are based on U.S. medical schools, and although the discussion briefly mentions other countries, external generalisability is limited.

6. Limited Representation of Gender-diverse Students:

Non-binary, intersex, and gender-diverse students were not included due to data limitations. This restricts inclusivity and the applicability of findings.

7. Reliance on Self-report:

Both exposures (discrimination) and outcomes (development) are based on self-report, which may be influenced by recall bias, perception differences, or social desirability bias.

8. Narrow Outcome Measures:

Personal and professional identity formation (PPIF) is reduced to two single items, which may not capture the multidimensional and dynamic nature of identity formation.

9. Limited Consideration of Intersectionality:

While race/ethnicity and income were adjusted for, the intersection of gender with race, sexual orientation, or socioeconomic status is not deeply explored. This may obscure important subgroup differences.

10. Potential Cohort Effect:

The study spans students from 2014–2020. Cultural changes over time (e.g., #MeToo movement, institutional reforms) may have influenced responses, but temporal trends are not examined.

11. Overinterpretation of Male vs. Female Differences:

The paper emphasizes that males showed sharper declines in PPIF with discrimination, but this interpretation may be speculative without qualitative evidence to support mechanisms.

12. Heavy Tables/Figures:

Some tables and figures are very dense and could be simplified or moved to supplementary material for clarity.

13. Policy Recommendations Could Be Sharper:

While the manuscript calls for reforms, the recommendations remain somewhat general (e.g., “implicit bias training”). More concrete, evidence-based interventions would strengthen the implications.

**********

7. PLOS authors have the option to publish the peer review history of their article (what does this mean?). If published, this will include your full peer review and any attached files.

If you choose “no”, your identity will remain anonymous but your review may still be made public.

**Do you want your identity to be public for this peer review?**   For information about this choice, including consent withdrawal, please see our Privacy Policy.

Reviewer #1: No

Reviewer #2: No

**********

[NOTE: If reviewer comments were submitted as an attachment file, they will be attached to this email and accessible via the submission site. Please log into your account, locate the manuscript record, and check for the action link "View Attachments". If this link does not appear, there are no attachment files.]

While revising your submission, please upload your figure files to the Preflight Analysis and Conversion Engine (PACE) digital diagnostic tool, https://pacev2.apexcovantage.com/. PACE helps ensure that figures meet PLOS requirements. To use PACE, you must first register as a user. Registration is free. Then, login and navigate to the UPLOAD tab, where you will find detailed instructions on how to use the tool. If you encounter any issues or have any questions when using PACE, please email PLOS at figures@plos.org. Please note that Supporting Information files do not need this step.

# Author comment: Author response to Decision Letter 2

**DOI:** 10.1371/journal.pone.0319549.r004

10 Nov 2025

Dear Dr. Ramagopalan,

Thank you for you review of our manuscript, “Gender Discrimination and Medical Student Development.” We appreciate the reviewers’ thoughtful comments and recommendations and have responded to each concern and suggestion below:

JOURNAL REQUIREMENTS:

1. If the reviewer comments include a recommendation to cite specific previously published works, please review and evaluate these publications to determine whether they are relevant and should be cited. There is no requirement to cite these works unless the editor has indicated otherwise.

Response: We reviewed all reviewer comments carefully. None included recommendations to cite specific previously published works.

REVIEWER’S COMMENTS

Reviewer #1:

1. I would like to thank the authors of the manuscript for making the requested corrections. It seems that the authors took a lot of time and care to make the corrections, which is appreciated. However, the following needs serious attention:

The authors stated regarding the validity and reliability of the instrument that "While not part of a validated scale, they exhibit strong face validity as direct, interpretable indicators of serious forms of gender discrimination in medical school settings."

If the instrument is not standardized, the manuscript and its data are not reliable. Were validity and reliability calculated for your study?

So I chose "Major Revision" to emphasize the importance of this issue.

Although these two tools were developed by a reputable and well-known scientific source, they need to be validated for your study population and context.

Response: We thank the reviewer for emphasizing the importance of measurement validity and reliability. We have now evaluated the internal consistency of the three AAMC Graduation Questionnaire (GQ) items assessing gender discrimination among respondents with complete data on all three items. Cronbach’s α = 0.58, indicating moderate inter-item coherence. This level of internal consistency is consistent with expectations for a brief, formative index designed to capture distinct manifestations of gender-based mistreatment (denied opportunities, sexist remarks, and biased evaluations). Because these items represent a formative rather than reflective construct, high inter-item correlations are not theoretically required.

Regarding validity, although no formal psychometric validation of these specific items has been published, they have been used consistently in multiple peer-reviewed national studies to assess gender discrimination and related outcomes among U.S. medical students (Nguyen et al., JAMA Pediatrics, 2022; Nguyen et al., JAMA Network Open, 2025). Their reproducible associations with key educational outcomes across diverse cohorts provide indirect evidence of construct validity. Moreover, the items are derived from a nationally administered instrument that undergoes annual expert review to ensure content relevance and clarity, supporting their face and content validity within this study context.

We have incorporated this clarification into the Methods section.

References:

Nguyen M, Chaudhry SI, Desai MM, Chen C, McDade WA, Fancher TL, Boatright D. Association of mistreatment and discrimination with medical school attrition. JAMA pediatrics. 2022 Sep 1;176(9):935-7.

Nguyen M, Venkataraman S, Abrams G, Pereira-Lima K, Fancher T, Addams AN, Moreland CJ, Boatright DH, Meeks LM. Discrimination Experiences Among Medical Students. JAMA Network Open. 2025 Oct 1;8(10):e2537871-.

Modified text (lines 199-206): “Internal consistency of the three gender discrimination items was assessed among respondents with complete data on all three items. Cronbach’s α = 0.58, indicating moderate inter-item coherence. This level of consistency is expected for a brief, three-item measure that captures distinct manifestations of gender-based mistreatment (denied opportunities, sexist remarks, and biased evaluations). Because these items represent a formative index rather than a reflective scale, high internal consistency is not theoretically required. The same AAMC GQ items have been used in multiple national studies examining discrimination among U.S. medical students. [11,12]”

Reviewer #2:

1. Sex vs. Gender Measurement:

The manuscript relies on a binary “sex” variable from AMCAS that may represent sex assigned at birth or gender identity. This ambiguity could introduce misclassification bias and should be more thoroughly emphasized.

Response: We agree and now emphasize the ambiguity of the AMCAS “Sex” item and its implications. We retain the term “sex” to match the instrument label but clarify in a clear and transparent manner that responses may reflect sex and/or gender identity in both the “Methods” and “Limitations” sections. We explicitly note potential nondifferential misclassification and its implication of biasing associations to the null.

Modified text (lines 168-173): “The item was labeled ‘Sex’ on the 2014 and 2015 AMCAS application and was not accompanied by a definition or clarification about whether it referred to sex assigned at birth or gender identity. As such, this variable may reflect sex, gender identity, or a combination thereof, depending on how applicants interpreted the question. We therefore interpret analyses as stratified by the AMCAS ‘Sex’ response and acknowledge potential nondifferential misclassification.”

Modified text (lines 474-484): “Although we refer to this variable as ‘sex’ to match the instrument label, the AMCAS item lacked a definition, which may have led some respondents to interpret it as gender identity, introducing potential misclassification that is likely nondifferential with respect to the outcomes and would bias associations toward the null. Prior work suggest that, in the absence of clarification, respondents may conflate sex and gender when completing survey items.[43] Similarly, the absence of sexual orientation data for our study years limits our ability to explore how intersecting sexual minority status (lesbian, gay, or bisexual identity), may influence personal and professional development. Together, these data limitations restrict the inclusivity and applicability of our findings and highlight the need for future research that captures a broader spectrum of sex, gender, and sexual orientation in medical education.”

2. Lack of Psychometric Validation:

The three AAMC Graduation Questionnaire items used to measure gender discrimination and the two items for personal/professional development have face validity but no established psychometric validation. This limits the reliability of the constructs.

Response: We added an internal-consistency check for the three GQ discrimination items (Cronbach’s α = 0.58 using respondents with complete data on all three items). We explain that this brief set functions as a formative index capturing distinct manifestations of gender discrimination; therefore, high α is not expected. For the PPIF items, we explicitly state that reliability coefficients are not applicable to single items, and that these items function as global indicators of perceived institutional support. We cite prior national studies employing the same discrimination and PPIF items.

Modified text (lines 199-206): “Internal consistency of the three gender discrimination items was assessed among respondents with complete data on all three items. Cronbach’s α = 0.58, indicating moderate inter-item coherence. This level of consistency is expected for a brief, three-item measure that captures distinct manifestations of gender-based mistreatment (denied opportunities, sexist remarks, and biased evaluations). Because these items represent a formative index rather than a reflective scale, high internal consistency is not theoretically required. The same AAMC GQ items have been used in multiple national studies examining discrimination among U.S. medical students.[11,12]”

Modified text (lines 218-221): “While they are not components of a validated scale and cannot be tested for reliability as single items, they are used in national research and institutional evaluation as global indicators of perceived institutional support for personal and professional development.[14,15]”

3. Cross-sectional Design:

The retrospective cross-sectional design precludes causal inference. The possibility of reverse causality (e.g., lower development perceptions influencing how students interpret discrimination) is not fully addressed.

Response: We thank the reviewer for this astute observation. We agree that we cannot infer causality and have now moreover clarified that temporal ordering cannot be established; perceptions of development could plausibly shape reporting of discrimination. We interpret estimates as associations consistent with theory and prior qualitative work and call for longitudinal mixed-methods.

Modified text (lines 505-508): “Finally, given the retrospective cross-sectional design, temporal ordering cannot be established, and reverse causality is possible (e.g., lower perceived development could influence interpretation/reporting of discrimination experiences). Associations should therefore be interpreted as non-causal.”

4. High Missing Data and Imputation:

Key variables (income, discrimination, outcomes) had large amounts of missing data (up to ~27%), and multiple imputation was used. While sensitivity analyses are reported, more discussion of how missingness may bias results would strengthen the paper.

Response: We thank the reviewer for raising this important point. We’ve now expanded the description of our multiple imputation strategy in the “Methods” section and added a statement on how missingness may bias results in the “Limitations” section. The imputation model included variables predictive of missingness and of the outcomes, and diagnostics compared observed versus imputed distributions. Complete-case results were directionally and numerically similar, reducing concern for bias from listwise deletion.

Modified text (lines 231-236): “The multiple imputation model included all predictors and outcomes as well as indicators plausibly related to missingness. Twenty imputed data sets were created. We assumed data were missing at random conditional on included variables. Diagnostics demonstrated model convergence and close alignment between observed and imputed marginal distributions (available on request). Complete-case analyses (n=21,728) yielded estimates similar in direction and magnitude (available on request).”

Modified text (lines 490-492): “Further, residual bias may persist if data were not missing at random. For instance, underrepresentation of students experiencing discrimination could lead to conservative estimates of its association with educational outcomes.”

5. Generalisability Outside the U.S.:

Findings are based on U.S. medical schools, and although the discussion briefly mentions other countries, external generalisability is limited.

Response: We agree and now state explicitly that external generalisability is limited in the discussion paragraph on “Applicability to other contexts”. This paragraph was originally added in response to prior reviewer feedback requesting consideration of this study’s relevance to other contexts and cultures in the “Discussion” section.

Modified text (lines 457-460): “Our study draws on data from U.S. medical schools and may have limited generalizability to medical educational settings outside North America where sociocultural norms, gender dynamics, and institutional structures differ and may shape both the experience and perceived impact of gender discrimination in distinct ways.

6. Limited Representation of Gender-diverse Students:

Non-binary, intersex, and gender-diverse students were not included due to data limitations. This restricts inclusivity and the applicability of findings.

Response: We agree and have further emphasized this limitation borne from AAMC data unavailability for our study years and proposed this line of enquiry as a future direction.

Modified text (lines 472-484): “Our binary sex variable (female/male) does not capture intersex variations. For our study years, the AAMC did not release data on intersex, non-binary, or other gender-diverse individuals because of the small sample sizes and concerns about maintaining anonymity. Although we refer to this variable as ‘sex’ to match the instrument label, the AMCAS item lacked a definition, which may have led some respondents to interpret it as gender identity, introducing potential misclassification that is likely nondifferential with respect to the outcomes and would bias associations toward the null. Prior work suggest that, in the absence of clarification, respondents may conflate sex and gender when completing survey items.[43] Similarly, the absence of sexual orientation data for our study years limits our ability to explore how intersecting sexual minority status (lesbian, gay, or bisexual identity), may influence personal and professional development. Together, these data limitations restrict the inclusivity and applicability of our findings and highlight the need for future research that captures a broader spectrum of sex, gender, and sexual orientation in medical education.”

7. Reliance on Self-report:

Both exposures (discrimination) and outcomes (development) are based on self-report, which may be influenced by recall bias, perception differences, or social desirability bias.

Response: We appreciate these important points and have now explicitly acknowledged the stated biases associated with self-reported data.

Modified text (lines 503-505): “Moreover, as our outcomes and exposures rely on self-report, recall bias, sex differences in perception or interpretation, and social desirability bias may influence reporting patterns.”

8. Narrow Outcome Measures:

Personal and professional identity formation (PPIF) is reduced to two single items, which may not capture the multidimensional and dynamic nature of identity formation.

Response: We agree and now explicitly acknowledge this limitation.

Modified text (lines 497-500): “Each outcome was derived from a single survey item that, while used nationally as a global indicator of institutional support, is not part of a validated multidimensional scale. Consequently, these measures may not encompass the complex, dynamic, and individualized nature of personal and professional identity formation.”

9. Limited Consideration of Intersectionality:

While race/ethnicity and income were adjusted for, the intersection of gender with race, sexual orientation, or socioeconomic status is not deeply explored. This may obscure important subgroup differences.

Response: We appreciate this thoughtful comment and agree that intersectional approaches are essential for understanding how multiple systems of marginalization jointly shape students’ experiences and development. Our primary aim in this study was to examine associations between gender discrimination and identity formation, and intersectional analyses, though important, were beyond the current scope. To acknowledge this point, we revised the opening of the “Implications” section to emphasize the need for future work that investigates how gender discrimination interacts with other axes of marginalization.

Modified text (lines 510-514): “Our findings highlight a persisting need for systemic reforms in medical education to address gender discrimination that is experienced by both female and male students, and to expand inquiry into how such discrimination intersects with broader axes of marginalization—rooted in racialization, class, and sexuality—to shape personal and professional identity formation and educational trajectories, including among gender-diverse students.”

10. Potential Cohort Effect:

The study spans students from 2014–2020. Cultural changes over time (e.g., #MeToo movement, institutional reforms) may have influenced responses, but temporal trends are not examined.

Response: We appreciate this comment and agree that cultural and institutional changes during the study period could have contributed to cohort differences. To account for potential temporal variation, we re-estimated all models including

Attachment Submitted filename: Response to Reviewers_Nov 10.docx

# Peer review: Decision Letter 2

**DOI:** 10.1371/journal.pone.0319549.r005

**Authors:** Somayeh Delavari, Sreeram Ramagopalan, Sreeram Ramagopalan

9 Feb 2026

PONE-D-25-05663R2

Gender Discrimination and Medical Student Development

PLOS One

Dear Dr. Venkataraman,

Thank you for submitting your manuscript to PLOS ONE. After careful consideration, we feel that it has merit but does not fully meet PLOS ONE’s publication criteria as it currently stands. Therefore, we invite you to submit a revised version of the manuscript that addresses the points raised during the review

Please submit your revised manuscript by Mar 26 2026 11:59PM. If you will need more time than this to complete your revisions, please reply to this message or contact the journal office at plosone@plos.org. When you're ready to submit your revision, log on to https://www.editorialmanager.com/pone/ and select the 'Submissions Needing Revision' folder to locate your manuscript file.

Please include the following items when submitting your revised manuscript:

- A letter that responds to each point raised by the academic editor and reviewer(s). You should upload this letter as a separate file labeled 'Response to Reviewers'.
- A marked-up copy of your manuscript that highlights changes made to the original version. You should upload this as a separate file labeled 'Revised Manuscript with Track Changes'.
- An unmarked version of your revised paper without tracked changes. You should upload this as a separate file labeled 'Manuscript'.

If you would like to make changes to your financial disclosure, please include your updated statement in your cover letter. Guidelines for resubmitting your figure files are available below the reviewer comments at the end of this letter.

If applicable, we recommend that you deposit your laboratory protocols in protocols.io to enhance the reproducibility of your results. Protocols.io assigns your protocol its own identifier (DOI) so that it can be cited independently in the future. For instructions see: https://journals.plos.org/plosone/s/submission-guidelines#loc-laboratory-protocols. Additionally, PLOS ONE offers an option for publishing peer-reviewed Lab Protocol articles, which describe protocols hosted on protocols.io. Read more information on sharing protocols at https://plos.org/protocols?utm_medium=editorial-email&utm_source=authorletters&utm_campaign=protocols.

We look forward to receiving your revised manuscript.

Kind regards,

Sreeram V. Ramagopalan

Academic Editor

PLOS One

Journal Requirements:

If the reviewer comments include a recommendation to cite specific previously published works, please review and evaluate these publications to determine whether they are relevant and should be cited. There is no requirement to cite these works unless the editor has indicated otherwise.

[Note: HTML markup is below. Please do not edit.]

Reviewers' comments:

Reviewer's Responses to Questions

**Comments to the Author**

1. If the authors have adequately addressed your comments raised in a previous round of review and you feel that this manuscript is now acceptable for publication, you may indicate that here to bypass the “Comments to the Author” section, enter your conflict of interest statement in the “Confidential to Editor” section, and submit your "Accept" recommendation.

Reviewer #1: (No Response)

Reviewer #3: (No Response)

**********

2. Is the manuscript technically sound, and do the data support the conclusions?

The manuscript must describe a technically sound piece of scientific research with data that supports the conclusions. Experiments must have been conducted rigorously, with appropriate controls, replication, and sample sizes. The conclusions must be drawn appropriately based on the data presented.

Reviewer #1: No

Reviewer #3: Partly

**********

3. Has the statistical analysis been performed appropriately and rigorously?

Reviewer #1: No

Reviewer #3: No

**********

4. Have the authors made all data underlying the findings in their manuscript fully available?

The PLOS Data policy requires authors to make all data underlying the findings described in their manuscript fully available without restriction, with rare exception (please refer to the Data Availability Statement in the manuscript PDF file). The data should be provided as part of the manuscript or its supporting information, or deposited to a public repository. For example, in addition to summary statistics, the data points behind means, medians and variance measures should be available. If there are restrictions on publicly sharing data—e.g. participant privacy or use of data from a third party—those must be specified.

Reviewer #1: Yes

Reviewer #3: Yes

**********

5. Is the manuscript presented in an intelligible fashion and written in standard English?

PLOS ONE does not copyedit accepted manuscripts, so the language in submitted articles must be clear, correct, and unambiguous. Any typographical or grammatical errors should be corrected at revision, so please note any specific errors here.

Reviewer #1: Yes

Reviewer #3: Yes

**********

6. Review Comments to the Author

Please use the space provided to explain your answers to the questions above. You may also include additional comments for the author, including concerns about dual publication, research ethics, or publication ethics. (Please upload your review as an attachment if it exceeds 20,000 characters)

Reviewer #1: Dear colleague

The data provided about the questionnaire does not confirm its reliability and validity.

In fact, gender discrimination was examined with three questions that have no quantitative and qualitative support (validity confirmation). The authors considered its validity and reliability to be confirmed because the questionnaire was developed by AAMC.

These issues make the value of the data problematic. Cronbach's alpha indicates the reliability of the data, and factor analysis is needed to examine the validity. How were these three questions extracted?

Also, two questions (one question related to personal development and one question related to professional development) were used to assess personal and professional development. While there are standard tools for assessing professional identity, two questions alone are not a complete criterion for assessing professional identity.

In other words, the relationship between different questions in a questionnaire was used to examine the two variables of gender discrimination and professional identity.

The study analyzed data between 2015 and 2020, while we are now in 2026, and many factors have affected the two variables above. Given the changing academic generations, the above conclusion may be affected.

It would have been reliable and valuable if the researchers had first examined the psychometrics of the questionnaire, identified the factors, and then examined this issue in graduates of different years

Reviewer #3: The manuscript could be strengthened with further refinement.

Line 225: The total percentage missing data to be stated.

Line 229: For the sentence ‘Missing data were imputed using a fully conditional specification method to handle arbitrary missing patterns across all categorical data.', what about the continuous data and what sort of statistical approach was used?

Line 252-253: The statement ‘Sensitivity analyses including indicator terms for GQ year yielded results consistent with the main models, suggesting minimal influence of cohort effects.’ is to be placed in the results section. If were to place in the methods section, it should state ‘Sensitivity analyses including indicator terms for GQ year were conducted to assess potential cohort effects.’

Line 233: MAR is to be placed in bracket for missing at random.

All statistical tests used in this study are to be reported in the Statistical Analyses section.

Table 1: 'Other' is to be placed after ‘White’ category.

Statistical results and figures in the Results section are to be presented in tables to provide detailed information and to facilitate comparison and interpretation. Some tables may be included as supplementary files.

The detailed table output including interaction term derived from GLM is to be presented.

**********

7. PLOS authors have the option to publish the peer review history of their article (what does this mean?). If published, this will include your full peer review and any attached files.

If you choose “no”, your identity will remain anonymous but your review may still be made public.

**Do you want your identity to be public for this peer review?**   For information about this choice, including consent withdrawal, please see our Privacy Policy.

Reviewer #1: No

Reviewer #3: No

**********

[NOTE: If reviewer comments were submitted as an attachment file, they will be attached to this email and accessible via the submission site. Please log into your account, locate the manuscript record, and check for the action link "View Attachments". If this link does not appear, there are no attachment files.]

To ensure your figures meet our technical requirements, please review our figure guidelines: https://journals.plos.org/plosone/s/figures

You may also use PLOS’s free figure tool, NAAS, to help you prepare publication quality figures: https://journals.plos.org/plosone/s/figures#loc-tools-for-figure-preparation.

NAAS will assess whether your figures meet our technical requirements by comparing each figure against our figure specifications.

# Author comment: Author response to Decision Letter 3

**DOI:** 10.1371/journal.pone.0319549.r006

27 Mar 2026

REVIEWER’S COMMENTS

Reviewer #1:

1. The data provided about the questionnaire does not confirm its reliability and validity. In fact, gender discrimination was examined with three questions that have no quantitative and qualitative support (validity confirmation). The authors considered its validity and reliability to be confirmed because the questionnaire was developed by AAMC. These issues make the value of the data problematic. Cronbach's alpha indicates the reliability of the data, and factor analysis is needed to examine the validity. How were these three questions extracted?

Response: The three gender-discrimination items used in our study are part of the AAMC’s Graduation Questionnaire (GQ), and these exact items were incorporated into the validated PRODIGIE tool (Boatright et al., 2024). As shown in Boatright et al. (2024), these GQ discrimination items underwent exploratory factor analysis, confirmatory factor analysis, and internal-consistency testing within a national dataset of over 60,000 medical students. In the PRODIGIE validation, the GQ discrimination factor (“Discrimination: race, ethnicity, and gender”) demonstrated acceptable psychometric performance with factor loadings ≥0.43 and Cronbach’s α = 0.76 (Boatright et al., 2024). Thus, the validity and reliability of these items have been established in prior psychometric evaluation. In our manuscript, we additionally reported Cronbach’s α for these three items gender discrimination items in our dataset, as previously requested.

Reference: Boatright, D., Nguyen, M., Hill, K., Berg, D., Castillo-Page, L., Anderson, N., ... & White, M. A. (2024). Development of a tool to measure student perceptions of equity and inclusion in medical schools. JAMA Network Open, 7(2), e240001.

Modified text (lines 195-203): “These items have been psychometrically validated as part of the PRODIGIE tool using the same 2016–2020 GQ cohorts analyzed in our study, undergoing exploratory and confirmatory factor analyses and demonstrating acceptable internal consistency for the discrimination factor (Cronbach α = 0.76).[11] While these items were validated within the broader discrimination factor in PRODIGIE, we use them here as a formative index capturing distinct manifestations of gender‑based mistreatment, for which high internal consistency is not required. When assessed in our sample, the three items demonstrated a Cronbach’s α of 0.58, which is expected for a brief formative index.”

2. Also, two questions (one question related to personal development and one question related to professional development) were used to assess personal and professional development. While there are standard tools for assessing professional identity, two questions alone are not a complete criterion for assessing professional identity. In other words, the relationship between different questions in a questionnaire was used to examine the two variables of gender discrimination and professional identity.

Response: We agree that the two GQ items used to assess personal and professional development are not part of a validated multi‑item scale and that they are not a complete criterion for assessing professional identity formation and have acknowledged this as a limitation (see lines 508-517). These items are single‑item indicators that are used nationally by the AAMC for institutional assessment and have been used in prior peer‑reviewed research for this purpose (Venkataraman et al., 2024, Venkataraman et al., 2026). Because they are single items, psychometric analyses such as internal consistency or factor analysis are not applicable. In the manuscript, we analyze these items separately and do not treat them as a scale, consistent with prior studies using these same GQ items. We have now amended the text, including the title of the manuscript, to make it clear that personal and professional development are two separate constructs that were measured separately.

References:

1) Venkataraman, S., Nguyen, M., Chaudhry, S. I., Desai, M. M., Hajduk, A. M., Mason, H. R., ... & Boatright, D. (2024). Racial and ethnic discrimination and medical students’ identity formation. JAMA Network Open, 7(10), e2439727.

2) Venkataraman, S., Nguyen, M., Hajduk, A. M., Ayedun, A., Roberts, W., Shanab, B., ... & Boatright, D. (2026). Meaningful Interactional Diversity, Professional Development, and Service Intent in White Medical Students. JAMA Network Open, 9(2), e2560266.

Modified text (line 1): “Gender discrimination and personal and professional development fostered by medical schools”

Modified text (lines 48-50): “PPIF was assessed using two separate GQ metrics assessing student agreement on a 5-point Likert scale that their medical school fostered and nurtured their development as a person and a future physician, respectively, and dichotomized.”

Associated text (line 206): “PPIF was assessed using two distinct metrics: personal and professional development.”

Associated text (lines 213-215): “Both personal and professional development were examined separately in all analyses; the term PPIF is used to collectively refer to these two outcomes.”

3. The study analyzed data between 2015 and 2020, while we are now in 2026, and many factors have affected the two variables above. Given the changing academic generations, the above conclusion may be affected.

Response: The study period (2016–2020) reflects the years for which all required GQ variables were available in analyzable form when this study was conducted. Because the reviewer’s concern relates to potential changes across academic years, we evaluated this directly by conducting sensitivity analyses that included indicator terms for GQ year. These analyses produced estimates that were highly consistent with the main models, indicating that associations did not vary meaningfully across cohorts in the available data. Nonetheless, we acknowledge that patterns should be reexamined in more recent cohorts, and have noted this as a future direction.

Modified text (lines 579-583): “Future research is needed to understand how gender discrimination influences the holistic development of medical students across the spectrum of gender identity and expression—including in more recent national cohorts—and through longitudinal qualitative and mixed methods approaches that include women, men, and gender-diverse students with intersecting marginalized identities.”

4. It would have been reliable and valuable if the researchers had first examined the psychometrics of the questionnaire, identified the factors, and then examined this issue in graduates of different years

Response: The discrimination items used in our study have already undergone psychometric evaluation in prior work (please see response to Comment #1). As part of the PRODIGIE validation, these same GQ discrimination items were examined using the 2016–2020 AAMC Graduation Questionnaire cohorts—the identical GQ years included in our study—and underwent exploratory and confirmatory factor analyses as well as internal‑consistency testing (Boatright et al., 2024). Because these items have already been validated using the same national GQ administrations, additional psychometric analyses were not required for the present study.

Regarding the reviewer’s point about “graduates of different years,” we evaluated potential cohort differences directly by conducting sensitivity analyses that included indicator terms for GQ year. These analyses produced estimates consistent with the main models, indicating no evidence of cohort‑related variation in the associations for the years examined.

Reviewer #3:

1. Line 225: The total percentage missing data to be stated.

Response: We have now added this information to the ‘Methods’ section.

Modified text (lines 226-227): “A total of 21,728 participants (57.77%) answered all the questionnaire items relevant to this study.”

2. Line 229: For the sentence ‘Missing data were imputed using a fully conditional specification method to handle arbitrary missing patterns across all categorical data.', what about the continuous data and what sort of statistical approach was used?

Response: There were no continuous variables included in this analysis.

3. Line 252-253: The statement ‘Sensitivity analyses including indicator terms for GQ year yielded results consistent with the main models, suggesting minimal influence of cohort effects.’ is to be placed in the results section. If were to place in the methods section, it should state ‘Sensitivity analyses including indicator terms for GQ year were conducted to assess potential cohort effects.’

Response: The phrasing has been changed as advised in the ‘Methods’ section, and a statement of the results of the sensitivity analyses was included in the ‘Results’ section.

Modified text (lines 250-251): “Sensitivity analyses including indicator terms for GQ year were conducted to assess potential cohort effects.”

Modified text (lines 356-357): “Across analyses, adding GQ-year indicator terms in sensitivity analyses produced results consistent with the main models.”

4. Line 233: MAR is to be placed in bracket for missing at random.

Response: This addition was made.

Modified text (lines 230-231): “We assumed data were missing at random (MAR) conditional on included variables.”

5. All statistical tests used in this study are to be reported in the Statistical Analyses section.

Response: We have ensured that all statistical tests used in this study are reported in the “Statistical Analysis” subsection of the “Methods” section.

6. Table 1: 'Other' is to be placed after ‘White’ category.

Response:; The proposed change has now been made, however, please note that the groups are no longer listed in alphabetical order.

Modified text (line 258)): Please see Table 1.

7. Statistical results and figures in the Results section are to be presented in tables to provide detailed information and to facilitate comparison and interpretation. Some tables may be included as supplementary files.

Response: Thank you for this helpful suggestion. In response, we have created a complete set of detailed tables corresponding to each results figure. These tables provide all underlying numerical values, including Ns, percentages, and adjusted relative risks with 95% confidence intervals. As recommended, we have included these tables as Supporting Information (S1–S8 Tables), with each table clearly linked to the figure it corresponds to (e.g., “corresponds to Figure 3”).

8. The detailed table output including interaction term derived from GLM is to be presented.

Response: We have added the full generalized linear model output for both outcomes. The S9 Table (personal development) and S10 Table (professional development) report all coefficients from the Poisson GLM, including the sex × gender‑discrimination interaction terms, main‑effect terms, and all covariates. Each table includes the IRR, robust standard error, z‑statistic, p‑value, and 95% confidence interval, providing the exact interaction parameters estimated by the model.

Attachment Submitted filename: Response_to_Reviewers_auresp_3.docx

# Peer review: Decision Letter 3

**DOI:** 10.1371/journal.pone.0319549.r007

**Authors:** Somayeh Delavari, Sreeram Ramagopalan, Sreeram Ramagopalan, Sreeram Ramagopalan

9 Apr 2026

PONE-D-25-05663R3

Gender Discrimination and Personal and Professional Development Fostered by Medical Schools

PLOS One

Dear Dr. Venkataraman,

Thank you for submitting your manuscript to PLOS ONE. After careful consideration, we feel that it has merit but does not fully meet PLOS ONE’s publication criteria as it currently stands. Therefore, we invite you to submit a revised version of the manuscript that addresses the points raised during the review process.

Please submit your revised manuscript by May 24 2026 11:59PM. If you will need more time than this to complete your revisions, please reply to this message or contact the journal office at plosone@plos.org. When you're ready to submit your revision, log on to https://www.editorialmanager.com/pone/ and select the 'Submissions Needing Revision' folder to locate your manuscript file.

Please include the following items when submitting your revised manuscript:

- A letter that responds to each point raised by the academic editor and reviewer(s). You should upload this letter as a separate file labeled 'Response to Reviewers'.
- A marked-up copy of your manuscript that highlights changes made to the original version. You should upload this as a separate file labeled 'Revised Manuscript with Track Changes'.
- An unmarked version of your revised paper without tracked changes. You should upload this as a separate file labeled 'Manuscript'.

If you would like to make changes to your financial disclosure, please include your updated statement in your cover letter. Guidelines for resubmitting your figure files are available below the reviewer comments at the end of this letter.

If applicable, we recommend that you deposit your laboratory protocols in protocols.io to enhance the reproducibility of your results. Protocols.io assigns your protocol its own identifier (DOI) so that it can be cited independently in the future. For instructions see: https://journals.plos.org/plosone/s/submission-guidelines#loc-laboratory-protocols. Additionally, PLOS ONE offers an option for publishing peer-reviewed Lab Protocol articles, which describe protocols hosted on protocols.io. Read more information on sharing protocols at https://plos.org/protocols?utm_medium=editorial-email&utm_source=authorletters&utm_campaign=protocols.

As the corresponding author, your ORCID iD is verified in the submission system and will appear in the published article. PLOS supports the use of ORCID, and we encourage all coauthors to register for an ORCID iD and use it as well. Please encourage your coauthors to verify their ORCID iD within the submission system before final acceptance, as unverified ORCID iDs will not appear in the published article. *Only*   the individual author can complete the verification step; PLOS staff *cannot*   verify ORCID iDs on behalf of authors.

We look forward to receiving your revised manuscript.

Kind regards,

Sreeram V. Ramagopalan

Academic Editor

PLOS One

Journal Requirements:

If the reviewer comments include a recommendation to cite specific previously published works, please review and evaluate these publications to determine whether they are relevant and should be cited. There is no requirement to cite these works unless the editor has indicated otherwise.

Please review your reference list to ensure that it is complete and correct. If you have cited papers that have been retracted, please include the rationale for doing so in the manuscript text, or remove these references and replace them with relevant current references. Any changes to the reference list should be mentioned in the rebuttal letter that accompanies your revised manuscript. If you need to cite a retracted article, indicate the article’s retracted status in the References list and also include a citation and full reference for the retraction notice.

[Note: HTML markup is below. Please do not edit.]

Reviewers' comments:

Reviewer's Responses to Questions

**Comments to the Author**

1. If the authors have adequately addressed your comments raised in a previous round of review and you feel that this manuscript is now acceptable for publication, you may indicate that here to bypass the “Comments to the Author” section, enter your conflict of interest statement in the “Confidential to Editor” section, and submit your "Accept" recommendation.

Reviewer #3: All comments have been addressed

**********

2. Is the manuscript technically sound, and do the data support the conclusions?

The manuscript must describe a technically sound piece of scientific research with data that supports the conclusions. Experiments must have been conducted rigorously, with appropriate controls, replication, and sample sizes. The conclusions must be drawn appropriately based on the data presented.

Reviewer #3: Partly

**********

3. Has the statistical analysis been performed appropriately and rigorously?

Reviewer #3: No

**********

4. Have the authors made all data underlying the findings in their manuscript fully available?

The PLOS Data policy requires authors to make all data underlying the findings described in their manuscript fully available without restriction, with rare exception (please refer to the Data Availability Statement in the manuscript PDF file). The data should be provided as part of the manuscript or its supporting information, or deposited to a public repository. For example, in addition to summary statistics, the data points behind means, medians and variance measures should be available. If there are restrictions on publicly sharing data—e.g. participant privacy or use of data from a third party—those must be specified.

Reviewer #3: Yes

**********

5. Is the manuscript presented in an intelligible fashion and written in standard English?

PLOS ONE does not copyedit accepted manuscripts, so the language in submitted articles must be clear, correct, and unambiguous. Any typographical or grammatical errors should be corrected at revision, so please note any specific errors here.

Reviewer #3: Yes

**********

6. Review Comments to the Author

Please use the space provided to explain your answers to the questions above. You may also include additional comments for the author, including concerns about dual publication, research ethics, or publication ethics. (Please upload your review as an attachment if it exceeds 20,000 characters)

Reviewer #3: Supplementary tables

1. Technically, the p-value cannot be zero; use 'p < ..' instead.

2. Exp(B) is to be replaced with 'OR'.

3. Use the format '95% CI (lower–upper)' instead of separate columns for '95% CI Lower Limit' and '95% CI Upper Limit'.

4. The tables columns width is to be adjusted.

**********

7. PLOS authors have the option to publish the peer review history of their article (what does this mean?). If published, this will include your full peer review and any attached files.

If you choose “no”, your identity will remain anonymous but your review may still be made public.

**Do you want your identity to be public for this peer review?**   For information about this choice, including consent withdrawal, please see our Privacy Policy.

Reviewer #3: No

**********

[NOTE: If reviewer comments were submitted as an attachment file, they will be attached to this email and accessible via the submission site. Please log into your account, locate the manuscript record, and check for the action link "View Attachments". If this link does not appear, there are no attachment files.]

To ensure your figures meet our technical requirements, please review our figure guidelines: https://journals.plos.org/plosone/s/figures

You may also use PLOS’s free figure tool, NAAS, to help you prepare publication quality figures: https://journals.plos.org/plosone/s/figures#loc-tools-for-figure-preparation.

NAAS will assess whether your figures meet our technical requirements by comparing each figure against our figure specifications.

# Author comment: Author response to Decision Letter 4

**DOI:** 10.1371/journal.pone.0319549.r008

24 May 2026

REVIEWER’S COMMENTS

Reviewer #3:

1. Supplementary tables: Technically, the p-value cannot be zero; use 'p < ..' instead.

Response: Thank you for this important flag. We’ve changed the p values in supplementary tables 7 to 10 to non-zero values. If the p value is less than 0.001, we’ve indicated it as such, rather report it incorrectly as zero.

Modified text: Please see p-value reporting in supplementary tables 7-10 (S7 Table to S10 Table).

2. Supplementary tables: Exp(B) is to be replaced with 'OR'.

Response: Given that our models used a Poisson distribution with robust standard errors (a method specifically chosen for the common, binary outcomes in our study, a context in which odd’s ratios [ORs] are known to overestimate the magnitude of association compared to relative risks; see Zou, 2004), the exponentiated coefficients from these regression models represent adjusted relative risks (aRRs) rather than ORs. As such, we have replaced the “exp(b)” with “aRR”. We have also cited the ‘Zou, 2004’ reference to the ‘Statistical Analysis’ section of the ‘Methods’.

Modified text: We have replaced “exp(b)” with “aRR” in supplementary tables 7 and 8 (S7 Table and S8 Table). We have also updated the effect measure column header from "IRR" to "aRR" in supplementary tables 9 and 10 (S9 Table and S10 Table) for consistency.

Modified text (lines 60, 347, 353, 357): We have replaced instances of “exp(b)” in the manuscript text with “aRR”.

Modified text (lines 239-240): “We used this method because PPIF outcomes were common. [19,20]”

Reference: Zou, G. (2004). A modified Poisson regression approach to prospective studies with binary data. American journal of epidemiology, 159(7), 702-706.

3. Supplementary tables: Use the format '95% CI (lower–upper)' instead of separate columns for '95% CI Lower Limit' and '95% CI Upper Limit'.

Response: We have now used the suggested format to report 95% CIs in supplementary tables 3 to 10.

Modified text: Please see the 95% CI reporting in supplementary tables 3 to 10 (S3 Table to S10 Table).

4. Supplementary tables: The tables columns width is to be adjusted.

Response: We have now adjusted the column widths of all the supplementary tables to ensure readability.

Modified text: Please see supplementary tables 1 to 10 (S1 Table to S10 Table).

Attachment Submitted filename: Response_to_Reviewers_auresp_4.docx

# Editor report: Decision Letter 4

**DOI:** 10.1371/journal.pone.0319549.r009

**Authors:** Somayeh Delavari, Sreeram Ramagopalan, Sreeram Ramagopalan, Sreeram Ramagopalan, Sreeram Ramagopalan

27 May 2026

<p>Gender Discrimination and Personal and Professional Development Fostered by Medical Schools

PONE-D-25-05663R4

Dear Dr. Venkataraman,

We’re pleased to inform you that your manuscript has been judged scientifically suitable for publication and will be formally accepted for publication once it meets all outstanding technical requirements.

Within one week, you’ll receive an e-mail detailing the required amendments. When these have been addressed, you’ll receive a formal acceptance letter and your manuscript will be scheduled for publication.

An invoice will be generated when your article is formally accepted. Please note, if your institution has a publishing partnership with PLOS and your article meets the relevant criteria, all or part of your publication costs will be covered. Please make sure your user information is up-to-date by logging into Editorial Manager at Editorial Manager® and clicking the ‘Update My Information' link at the top of the page. For questions related to billing, please contact billing support.

If your institution or institutions have a press office, please notify them about your upcoming paper to help maximize its impact. If they’ll be preparing press materials, please inform our press team as soon as possible -- no later than 48 hours after receiving the formal acceptance. Your manuscript will remain under strict press embargo until 2 pm Eastern Time on the date of publication. For more information, please contact onepress@plos.org.

Kind regards,

Sreeram V. Ramagopalan

Academic Editor

PLOS One

Additional Editor Comments (optional):

Reviewers' comments:

# Editor report: Acceptance letter

**DOI:** 10.1371/journal.pone.0319549.r010

**Authors:** Somayeh Delavari, Sreeram Ramagopalan, Sreeram Ramagopalan, Sreeram Ramagopalan, Sreeram Ramagopalan

PONE-D-25-05663R4

PLOS One

Dear Dr. Venkataraman,

I'm pleased to inform you that your manuscript has been deemed suitable for publication in PLOS One. Congratulations! Your manuscript is now being handed over to our production team.

At this stage, our production department will prepare your paper for publication. This includes ensuring the following:

* All references, tables, and figures are properly cited

* All relevant supporting information is included in the manuscript submission,

* There are no issues that prevent the paper from being properly typeset

You will receive further instructions from the production team, including instructions on how to review your proof when it is ready. Please keep in mind that we are working through a large volume of accepted articles, so please give us a few days to review your paper and let you know the next and final steps.

Lastly, if your institution or institutions have a press office, please let them know about your upcoming paper now to help maximize its impact. If they'll be preparing press materials, please inform our press team within the next 48 hours. Your manuscript will remain under strict press embargo until 2 pm Eastern Time on the date of publication. For more information, please contact onepress@plos.org.

You will receive an invoice from PLOS for your publication fee after your manuscript has reached the completed accept phase. If you receive an email requesting payment before acceptance or for any other service, this may be a phishing scheme. Learn how to identify phishing emails and protect your accounts at https://explore.plos.org/phishing.

If we can help with anything else, please email us at customercare@plos.org.

Thank you for submitting your work to PLOS ONE and supporting open access.

Kind regards,

PLOS ONE Editorial Office Staff

on behalf of

Dr. Sreeram V. Ramagopalan

Academic Editor

PLOS One
